# Constructing Cell-Specific Causal Networks of Individual Cells for Depicting Dynamical Biological Processes

**DOI:** 10.34133/research.0743

**Published:** 2025-06-27

**Authors:** Xinzhe Huang, Luonan Chen, Xiaoping Liu

**Affiliations:** ^1^ Key Laboratory of Systems Health Science of Zhejiang Province, School of Life Science, Hangzhou Institute for Advanced Study, University of Chinese Academy of Sciences, Hangzhou 310024, China.; ^2^ School of Mathematical Sciences and School of AI, Shanghai Jiao Tong University, Shanghai 200240, China.

## Abstract

Causal inference is crucial in biological research, as it enables the understanding of complex relationships and dynamic processes that drive cellular behavior, development, and disease. Within this context, gene regulatory network (GRN) inference serves as a key approach for understanding the molecular mechanisms underlying cellular function. Despite substantial advancements, challenges persist in GRN inference, particularly in dynamic rewiring, inferring causality, and context specificity. To tackle these issues, we present single cell-specific causal network (SiCNet), a novel causal network construction method that utilizes single-cell gene expression profiles and a causal inference strategy to construct molecular regulatory networks at a single-cell level. Additionally, SiCNet utilizes cell-specific network information to construct network outdegree matrix (ODM), enhancing the performance of cell clustering. It also enables the construction of context-specific GRNs to identify key regulators of fate transitions for diverse processes such as cellular reprogramming and development. Furthermore, SiCNet can delineate the intricate dynamic regulatory processes involved in development, providing deep insights into the mechanisms governing cellular transitions and the gene regulation across developmental stages.

## Introduction

The single-cell RNA sequencing (scRNA-seq) technology has revolutionized molecular biology by providing high-resolution, large-scale transcriptome data at the single-cell level [1,2]. With this technology, researchers can identify rare or novel cell types in tissues, study cell differentiation, and uncover the molecular mechanisms behind diseases with computational methods [3–5]. Many biological processes, such as transcriptional regulation and epigenetic modifications, involve molecular interactions among transcriptional products and regulatory elements. Deciphering these complex regulation relationships at a single-cell level is crucial for understanding cellular heterogeneity and uncovering the underlying mechanisms of biological functions and disease progression. Causal relationships, which are central to molecular biology, are key to understanding the underlying mechanisms of biological processes. Unlike correlations, causality can distinguish which gene is the cause and which gene is the effect [6]. Generally, the cause gene is defined as the regulator and the effect gene is defined as the target gene. This is particularly important in understanding disease mechanisms, identifying drug targets, and reconstructing gene regulatory networks (GRNs). By focusing on causality, researchers can develop more precise treatments, gain deeper insights into biological systems, and advance fields such as synthetic biology and personalized medicine [7,8].

The inference of GRNs has become one of the key approaches for elucidating molecular causality in biological systems. Most well-known methods inferring GRNs for single-cell data, such as GENIE3 [9], GRNBoost2 [10], and PIDC [11], are limited to construct GRNs at the cell type level, but not at the individual cell level. These methods fail to account for the heterogeneity among individual cells in a population. Recently, methods like SSN [12] and PSSN [13] have been proposed to construct relevance networks for individual cells. However, these methods primarily focus on correlations between genes, potentially missing causal relationships and more complex regulatory interactions.

In this paper, we proposed a novel method to construct causal networks for individual cells, referred to as the single cell-specific causal network (SiCNet), to address a challenge of inferring causal relationships from single-cell expression profile. By incorporating a statistical concept rooted in causal inference called CVP [14], we applied this causal inference framework to construct cell-specific causal networks. By analyzing cell-specific causal networks, we can identify key regulators and potential regulatory relationships in the context of cancer, shedding light on cancer progression. Furthermore, by integrating these cell-specific causal networks, we can represent the states of individual samples and detect critical states by calculating dynamic network biomarker (DNB) scores. Additionally, we aim to uncover regulatory processes involved in cell reprogramming and dynamic regulatory mechanisms during cellular differentiation, providing insights into factors associated with reprogramming efficiency and hematopoietic specification.

## Results

### Overview of SiCNet

**Fig. 1.**
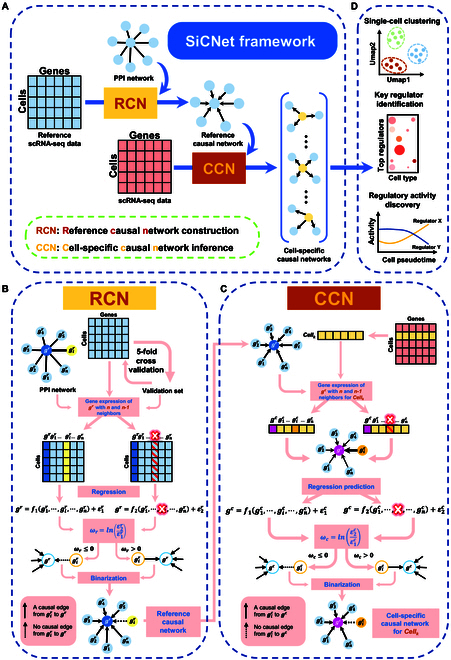
An overview of the SiCNet framework. (A) The SiCNet method takes reference scRNA-seq data and objective scRNA-seq data, along with protein–protein interaction (PPI) information, as inputs. It generates, as output, a set of causal networks for each cell. (B) Framework for inferring an initial reference causal network. (C) Framework for predicting single cell-specific causal networks. (D) The cell-specific networks generated as output are used for downstream analysis, including clustering, identifying key regulators, and discovering patterns of active regulators.

Based on the previous study [14], we used this causal network inference framework to develop a new approach, SiCNet, for predicting causal relationship at the single-cell level. The SiCNet is a data-driven and model-free algorithm for inferring cell-specific causal relationships between genes based on single-cell transcriptome (Fig. 1A).

To construct a causal network for a single cell, an additional single-cell transcriptomic dataset was required as a reference dataset. This reference dataset was used to establish an initial causal network based on the CVP method [14], and the initial causal network is as the reference causal network (Fig. 1B and Methods). Specifically, we employed 5-fold cross-validation to partition the reference dataset into 5 equal subsets. For each iteration, 4 subsets were used for model training, while the remaining subset served for model prediction. The causal inference was performed pairwise between genes. We trained 2 distinct regression models for each gene pair: one incorporating the presence of the cause gene, and another excluding it. The predictive performance of each model was evaluated by calculating the average root mean square error (RMSE), yielding $\varepsilon_{1}^{r}$ for models including the cause gene and $\varepsilon_{2}^{r}$ for those excluding it. The causal strength index (CSI; $\omega_{r}$) between the 2 genes was then quantified as the logarithm of the ratio between these RMSE values [$\ln\left(\varepsilon_{2}^{r}/\varepsilon_{1}^{r}\right)$]. A positive CSI ($\omega_{r}$ > 0) indicates a statistically significant causal effect between the gene pair, whereas a nonpositive value suggests the absence of such causal relationship (Fig. 1B and Methods). The subscript/superscript “*r*” in all symbols denotes the “reference”. The reference causal network captured general causal relationships between genes in the reference cells, serving as a benchmark of the initial GRN.

After obtaining the reference causal network, the gene expression data of a single cell were used to infer the causal relationships between genes (Fig. 1C and Methods). Specifically, for a target gene regulated by several other genes (regulators) in the reference network, we can predict the expression of this gene from the genes who regulate the target gene. Then, we can predict the target gene’s expression again after removing a regulating gene (regulator). If the prediction loss increases after removing a regulator, it indicates that the removed regulator has a causal influence on this target gene, thereby revealing its regulatory role in the causal network. Similarly, the causal relationship is determined by the sign of the CSI, which is represented by $\omega^{c}$ (Fig. 1C and Methods). The subscript/superscript “*c*” in all symbols denotes the “specific cell”. The cell-specific causal network was obtained by collecting regulation relationship of every gene, and it can reflect the individualized causal relationships adapted to the gene expression profile in particular cell. To enhance the reliability of the causal network prediction, a protein–protein interaction (PPI) network from the STRING database was used as the background network (Fig. 1 and Methods).

By identifying the set of target genes for each regulator, the regulatory activity of each gene can be quantified by counting the number of target genes in the cell-specific network. Based on this, a network outdegree matrix (ODM) was generated for further analysis (Fig. 1D and Methods). The ODM retains the same dimensions as the original gene expression matrix (GEM), with the same number of rows and columns, but it represents the higher-order regulatory information of each gene in the network. This matrix can be seamlessly analyzed using any standard scRNA-seq algorithm for tasks such as cell clustering, dimensionality reduction, and pseudo-trajectory analysis, simply by substituting the original GEM with our ODM.

### The performance of SiCNet in clustering on scRNA-seq datasets

To verify whether the SiCNet method can effectively characterize cellular heterogeneity, we constructed cell-specific networks for 5 scRNA-seq datasets (see Data Availability and Table S1) with explicit cellular annotations. For each dataset, we transformed the cell-specific networks constructed by SiCNet into an ODM for dimensionality reduction, visualization, and clustering analysis (see Methods).

**Fig. 2.**
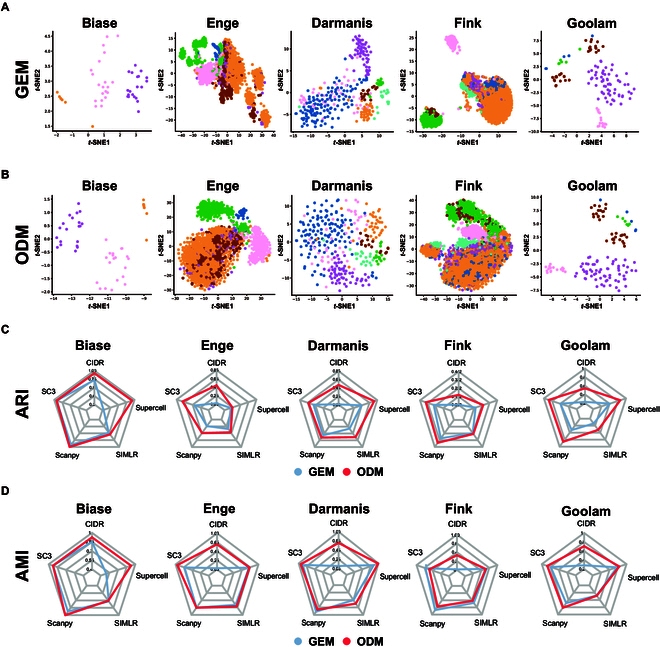
Five benchmark scRNA-Seq datasets demonstrated superior clustering performance with ODM compared to GEM over existing methods. (A) *t*-SNE visualization of GEM for the datasets. Different colors represent different cell types. (B) *t*-SNE visualization of ODM for the datasets. Different colors represent different cell types. (C) The ARI metrics are used to measure performance of clustering. Clustering methods SC3, CIDR, Scanpy, SIMLR, and Supercell are used to cluster datasets. (D) The AMI metrics are used to measure performance of clustering. Clustering methods SC3, CIDR, Scanpy, SIMLR, and Supercell are used to cluster datasets. GEM, gene expression matrix; ODM, network outdegree matrix; ARI, adjusted rand index; AMI, adjusted mutual information; *t*-SNE, *t*-distributed stochastic neighbor embedding.

For the purpose of visualizing the datasets, we employed *t*-distributed stochastic neighbor embedding (*t*-SNE) [15] to reduce the dimensionality of both the GEM and the ODM (Fig. 2A and B). Notably, the ODM visualizations exhibit clearer demarcation and clustering between different cell types, particularly in the Enge, Darmanis, and Fink datasets (Fig. 2B), where cell populations are more distinctly separated compared with the GEM visualizations (Fig. 2A). For example, in the Enge dataset, the representation of ODM clearly shows more compact and well-defined clusters, especially for cell types such as alpha (orange) and ductal (green), which are more tightly grouped compared to the GEM visualization (Fig. 2B). Similarly, in the Darmanis dataset, cell types such as oligodendrocyte precursor cells (OPCs) (cyan) and oligodendrocytes (orange) in ODM are more tightly grouped and clearly separated, while in the corresponding GEM visualization, these cell types reveal a more dispersed distribution with less defined separations (Fig. 2B). Overall, these results suggested that the ODM enhances the resolution and clarity of cell type distinctions, offering superior performance in terms of visualizing complex and high-dimensional data compared with the traditional GEM (Fig. 2B).

To quantitatively compare the clustering results of the ODM with the GEM, we applied 5 single-cell clustering algorithms, including SC3 [16], CIDR [17], Scanpy [18], SIMLR [19], and Supercell [20], across the 5 datasets. These clustering algorithms are widely used in single-cell analysis to uncover distinct cell populations. To evaluate the clustering performance, we calculated the adjusted rand index (ARI), adjusted mutual information (AMI), normalized mutual information (NMI), and Fowlkes–Mallows index (FMI) (Fig. 2C and D and Fig. S1). The results demonstrated that the representation of ODM outperformed the representation of GEM across most datasets, as evidenced by higher scores in the majority of clustering performance metrics. For example, in the Enge dataset, the ODM achieved higher ARI and AMI across all clustering algorithms, compared to the GEM (Fig. 2C and D). Specifically, ODM yielded an ARI of 0.58 and an AMI of 0.67 for the SC3 algorithm, compared to GEM’s ARI of 0.33 and AMI of 0.53. Similarly, for the SIMLR algorithm, ODM achieved an ARI of 0.35 and AMI of 0.51, while GEM showed an ARI of 0.27 and AMI of 0.47 (Fig. 2C and D). Similar trend was observed in the Darmanis and Goolam datasets, particularly with algorithms like Supercell and Scanpy (Fig. 2C and D). In the Darmanis dataset, ODM achieved an ARI of 0.63 and an AMI of 0.69 with Supercell, whereas GEM had an ARI of 0.37 and an AMI of 0.60. For the Scanpy algorithm, ODM reached an ARI of 0.47 and AMI of 0.59, outperforming GEM with an ARI of 0.42 and AMI of 0.63 (Fig. 2C and D). In the Goolam dataset, ODM demonstrated superior performance with an ARI of 0.86 and AMI of 0.83 for Supercell, compared to GEM’s ARI of 0.61 and AMI of 0.77. For Scanpy, ODM showed an ARI of 0.86 and AMI of 0.81, while GEM had an ARI of 0.53 and AMI of 0.69 (Fig. 2C and D). These results highlight the robustness and effectiveness of the ODM in uncovering distinct cell populations across diverse datasets.

### Revealing colorectal cancer progression patterns through SiCNet

**Fig. 3.**
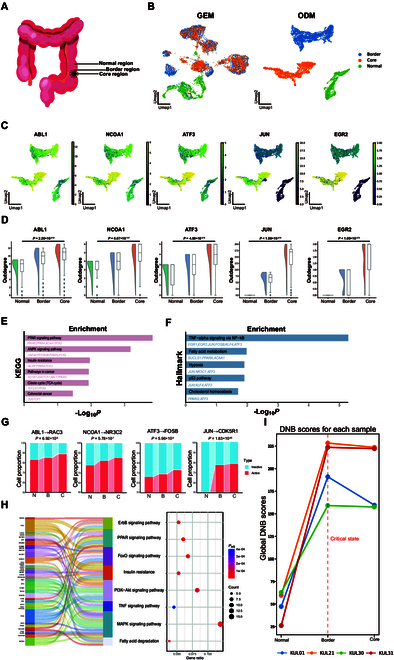
SiCNet reveals colorectal cancer progression regulatory patterns. (A) Diagram illustrating the colorectal, with the healthy adjacent tissues as well as the border and core regions of the tumor tissues. (B) Leiden clustering and UMAP visualization of GEM and ODM for the datasets. (C) UMAP visualization showing key regulators with high regulatory activity across the gradient from the healthy adjacent region to the core region. (D) Corresponding statistical analysis of the key regulators’ regulatory activity from the healthy adjacent region to the core region. *P* values were calculated using a 2-sided Student’s *t* test. (E) The identified regulators are significantly enriched in KEGG database to illustrate the progression of colorectal cancer. *P* values were calculated using hypergeometric test. (F) The identified regulators are significantly enriched in MSigDB database to illustrate the progression of colorectal cancer. *P* values were calculated using hypergeometric test. (G) Significantly up-regulated links found across 3 regions to illustrate the downstream regulation of colorectal cancer. *P* values were calculated using chi-square test. (H) Regulated genes from up-regulated links are significantly enriched in KEGG database to illustrate the downstream regulation of colorectal cancer. (I) Identification of critical states for cancer progression for each sample. KEGG, Kyoto Encyclopedia of Genes and Genomes; MSigDB, Molecular Signatures Database; UMAP, Uniform Manifold Approximation and Projection; GEM, gene expression matrix; ODM, network outdegree matrix.

To gain insight into the molecular mechanisms underlying disease at a single-cell network level, we obtained a human colorectal cancer scRNA-seq dataset [21], which includes cells from the healthy adjacent tissues as well as the border and core regions of the tumor tissues (Fig. 3A). It is worth noting that the colorectal cancer primarily originates from colon epithelial cells that lose genomic stability. This instability promotes the accumulation of mutations in tumor suppressor genes and oncogenes [22]. These mutations drive the malignant transformation of colon epithelial cells, leading to the formation of tumor core regions that expands outward through cycles of clonal expansion [23,24]. Therefore, in this study, we focused specifically on epithelial cells across 3 regions of tissue. The number of cells in the healthy tissue was 1,144. In the border region, the number of cells was 2,812, while in the core region, it was 2,212.

We applied the SiCNet method to construct cell-specific networks for each cell region and transformed these networks into 3 ODMs (see Methods): the ODM of the healthy adjacent region, the ODM of the border region, and the ODM of the core region. Notably, each of these ODMs includes the same number of genes as in the GEM and the corresponding number of cells specific to each region. Subsequently, we concatenated the 3 ODMs along the cell dimension, generating a single integrated ODM (6,168 cells) with all original genes retained. This new ODM consolidated information from the 3 regions. Finally, we performed dimensionality reduction and clustering on this new ODM matrix to uncover cancer progression patterns across the 3 regions. Compared with GEM, the ODM can better separate the cell populations of the border and core regions (Fig. 3B), further demonstrating that the ODM derived from the SiCNet method can effectively capture the characteristics and specificities of each cell. It is possible that the healthy adjacent tissues may gradually undergo malignant transformation at the tumor border region, with the lesions becoming more pronounced in the core region of the tumor. We performed a 2-sided Student’s *t* test on the ODM data between healthy adjacent and core regions to identify the key driver regulators of colorectal cancer progression (see Methods). For each regulator, the *P* value was adjusted for multiple testing using false discovery rate (FDR) correction (see Methods).

We finally identified 26 regulators with enhanced regulatory activity from the healthy adjacent region to the core region, aligning with previously reported oncogenic transformation patterns essential for the growth and development of colorectal tumors (Fig. 3C and D, Figs. S2 and S3, and Table S2). For example, the increased activity of *ABL1* (*P* = $2.20\times 10^{-219}$) is proved to be associated with decreased metastasis-free survival and/or lower overall survival in colorectal cancer [25,26]. The increased activity of *NCOA1* (*P* = $6.07\times 10^{-157}$) contributes to colorectal cancer progression by enhancing Hedgehog signaling [27]. The increased activity of *ATF3* (*P* = $4.89\times 10^{-278}$) drives colorectal cancer cell proliferation [28,29]. The increased activity of *JUN* (*P* < $1.00\times 10^{-278}$) triggers the activation of the c-Jun N-terminal kinase (JNK) and mitogen-activated protein kinase (MAPK) pathways in colorectal cancer [30,31]. The increased activity of *EGR2* (*P* < $1.00\times 10^{-278}$) facilitates the persistence of colorectal cancer stem cells [32]. In terms of gene expression, some genes, particularly *NCOA1* and *EGR2*, showed no significant differential expression between the healthy adjacent and core regions (Figs. S4 to S6 and Table S3).

To comprehensively evaluate the potential biological functions of these regulators, we conducted an enrichment analysis using the pathways from the Kyoto Encyclopedia of Genes and Genomes (KEGG) database and the hallmark gene sets from the Molecular Signatures Database (MSigDB). The KEGG enrichment analysis revealed that the regulators were enriched into the peroxisome proliferator-activated receptor (PPAR) signaling pathway, insulin resistance pathway, and the citrate cycle pathway by hypergeometric test with *P* values $1.49\times 10^{-4}$, $1.12\times 10^{-2}$, and $1.21\times 10^{-2}$ (Fig. 3E). All of these pathways are associated with the metabolic pathways and critical for the development of colorectal cancer [33]. These signaling pathways suggest that metabolic dysregulation is one of the major contributing factors in the development and progression of colorectal cancer. It is consistent with the existing knowledge [34–37]. The Hallmark analysis highlighted the enrichment of tumor necrosis factor-α (TNF-α) signaling and hypoxia signaling by hypergeometric test with *P* values $5.24\times 10^{-6}$ and $1.25\times 10^{-2}$ (Fig. 3F). This signaling indicated that the inflammatory processes were involved in colorectal cancer, and that the molecular interactions in the tumor microenvironment also played a critical role [38,39]. This is in line with previous reports suggesting that the evasion of tumor immune surveillance and low levels of oxygen support cancer cell survival, both of which are the features of the core region of colorectal cancer [40,41].

### Regulatory analysis and identifying critical states of colorectal cancer

We then counted the number of active and inactive regulatory connections—where active connections represent regulatory connections present and inactive connections indicate their absence—in each cell-specific network across the healthy, border, and core region, and identified 133 significant up-regulation links by chi-square test that may be associated with the cancer progression from the healthy adjacent region to the core region (Fig. 3G and Table S4). Among these significant links, several regulated genes (target genes) were found to play a crucial role in colorectal cancer (Fig. 3G). For instance, *RAC3* is regulated by *ABL1* (Fig. 3G), and this enhanced regulation has been shown to contribute to chemoresistance in colon cancer cells and support the maintenance of cancer cell stemness [42,43]. Additionally, *NR3C2* is regulated by *NCOA1* (Fig. 3G), and this intensified regulation activates the Wnt/β-catenin signaling pathway, enhancing the invasion of colon cancer cells [44]. *FOSB* is increasingly regulated by *ATF3* from the healthy adjacent region to the core region (Fig. 3G), and it plays a critical role in regulating inflammatory molecule expression, which influences the therapeutic effects of drugs on colorectal cancer [45]. Moreover, *CDK5R1* has been identified as a prognostic marker associated with poor survival in colorectal cancer [46]. We also performed the KEGG enrichment analysis to confirm the association between these regulated genes and the colorectal cancer, highlighting their roles in cancer-associated signaling pathways (Fig. 3H). Consistent with previous reports, the MAPK signaling pathway, phosphatidylinositol 3-kinase (PI3K)/AKT signaling pathway, and Wnt/β-catenin signaling pathway were found to play critical roles in the progression and development of colorectal cancer (Fig. 3H) [47]. In addition, metabolic reprogramming-related pathways, such as the ERBB and FOXO signaling pathways, were also significantly enriched (Fig. 3H), indicating that the dysregulation of these processes promote cancer growth [48–50]. These findings further support the existing evidence that metabolic dysregulation is a hallmark of colorectal cancer, and it can offer potential avenues for therapeutic interventions by targeting cancer cell metabolism.

DNB is a model to detect the early warning signal before disease onset [51] based on biological network. In this dataset, the border region represents the transitional zone where cells shift from a normal (healthy region) to a malignant state (core region), and it can be considered as a critical state from normal to disease. Therefore, we aggregated the cell-specific networks of each region to characterize each sample and used the *l*-DNB method [52] to detect critical states (Note S2). Undoubtedly, in most samples, the border region was indeed detected as a critical state (Fig. 3I and Fig. S7). This implied the potential of SiCNet method in applying the DNB approach to detect critical states.

Overall, our SiCNet method provides a cell-specific causal network perspective that effectively characterizes the progression of colorectal cancer in epithelial cells, strongly supporting our current understanding on the development of cancer.

### Uncovering key regulators during reprogramming

Cellular reprogramming is the process of converting differentiated cells back to a pluripotent state, which has significant implications in regenerative medicine and patient-specific disease modeling [53–56]. However, the regulatory mechanisms driving this process remain poorly understood.

**Fig. 4.**
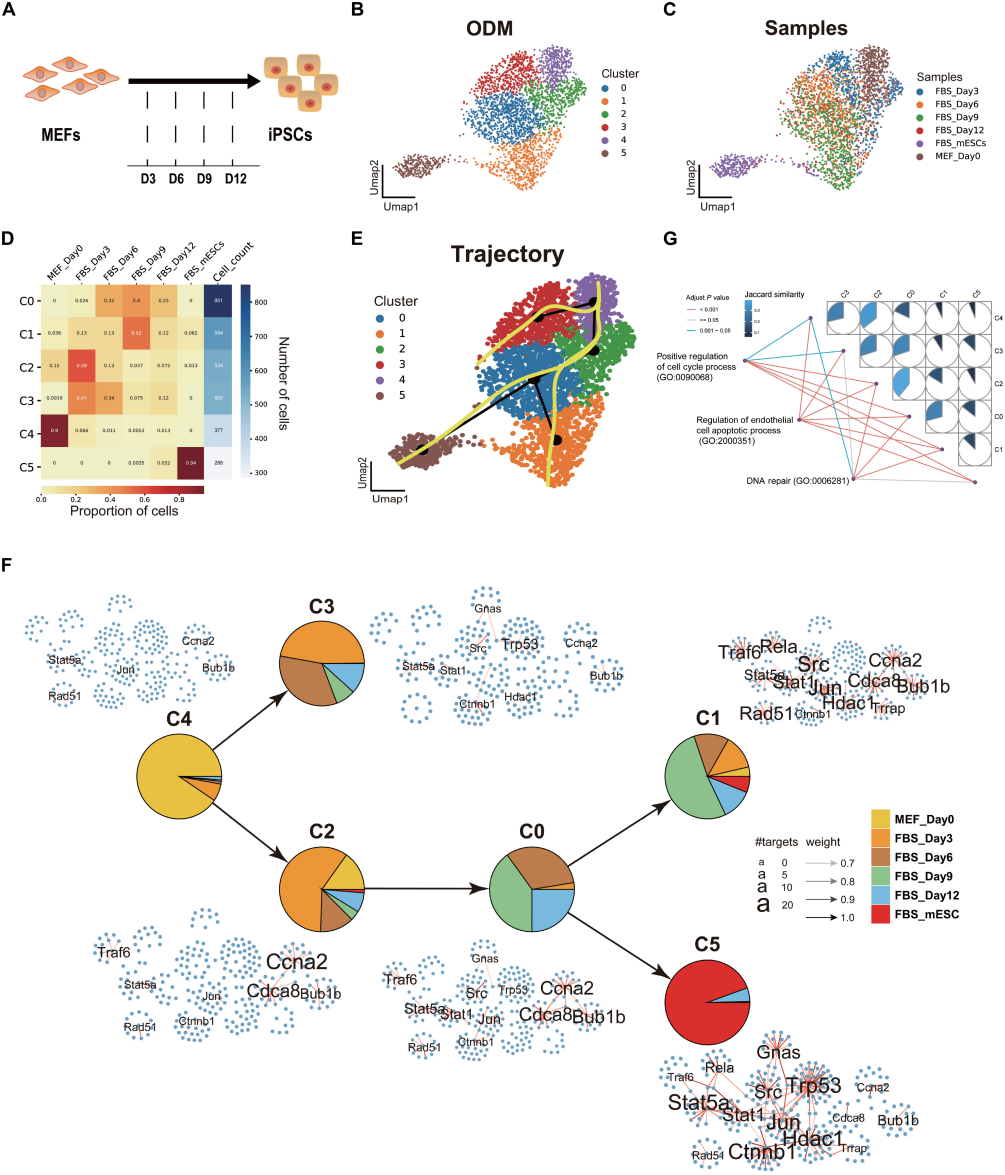
Constructing the cell type-specific networks to uncover the key regulators during reprogramming. (A) Diagram illustrating the mouse cellular reprogramming process and the sampling time point for the datasets. (B) UMAP of cell clusters on the ODM data of the data from mouse cellular reprogramming. (C) UMAP of cell clusters on the sample labels of the data from mouse cellular reprogramming. (D) Distribution of the sample labels in each cell cluster. (E) Inferred cell lineage trajectory for the 6 cell clusters with ODM data. (F) Cell lineage structure constructed through the inferred trajectory. The layout of each network is the same; edges present in a particular cell cluster are shown in red. Labeled nodes correspond to regulators with different targets. (G) Gene Ontology (GO) enrichment analysis on the regulators identified from each refined cell cluster-specific network. Jaccard similarity was calculated among these cell clusters with respective cell cluster-specific networks.

To gain deeper insights into this dynamic process, we gathered single-cell expression dataset capturing the reprogramming of mouse embryonic fibroblasts (MEFs) into pluripotent stem cells (mESCs). The dataset included 6 time points: the initial MEFs, fully reprogrammed mESCs, and intermediate stages spanning days 3 to 12 (days 3, 6, 9, and 12) (Fig. 4A). We applied the SiCNet method to construct cell-specific networks for the datasets with the 6 time points. These networks were then integrated into an ODM (see Methods). Using the Leiden clustering algorithm [57], we identified 6 distinct clusters (C0 to C5) (Fig. 4B). Each cluster represents a specific cell type in the reprogramming process. Obviously, based on the composition of cells from different time points within each cluster (Fig. 4C and D), C4 is MEF-specific, while C5 is mESC-specific, and the remaining clusters represented a mix of cells spanning different time points. C4 represents the starting cell type of reprogramming, C5 represents the end cell type, and the remaining clusters represent intermediate cell types of the process. This pattern, where cells from different time points are mixed within the clusters, reflects the inherent heterogeneity of the reprogramming system, consistent with its dynamics and stochasticity [58,59].

For each cluster, we calculated the proportion of active regulatory links (edges) in cell-specific networks that appeared in the cells of that cluster. Specifically, the proportion of an edge is defined as the number of cells containing the edge divided by the total number of cells in the cluster. Subsequently, we applied a cutoff to remove edges that were present in less than 70% of the cells in the cluster. This filtering step ensured that the retained regulatory interactions were consistently present in the majority of cells within the cluster. As a result, these retained regulatory interactions in each cluster formed a cell type-specific network. Among these networks, we identified the top transcriptional regulators associated with each of the cell clusters. For example, the MEF-specific cluster (C4) is enriched in *Hoxb4*, *Hoxb7*, *Dlx1*, and *Sox2*, all of which are related to development and differentiation processes [60–63]. In contrast, the mESC-specific cluster (C5) is characterized by the enrichment of *Stat5a*, *Smad3*, *Trp53*, and *Sin3a*, which play essential roles in maintaining pluripotency and regulating reprogramming [64–67]. Based on the earlier designation of the MEF-specific cluster (C4) as the starting point and the mESC-specific cluster (C5) as the endpoint of the reprogramming process, we employed the Slingshot approach [68] to infer the cellular trajectories across the 6 clusters (Fig. 4E).

To investigate the regulatory mechanisms during the reprogramming process, we focused on the key components of each cell type-specific network and compared the differences in regulatory networks across cell types (clusters). As the first key transition point (Fig. 4F), C2 contains the regulators such as *Ccna2*, *Cdca8*, and *Bub1b*, which are known to be involved in cell cycle progression (Fig. 4F). In contrast, these regulators are less prevalent or absent in C3. Consequently, C2 displays a higher cell cycle-related regulatory activity compared to C3. This distinct pattern of regulatory activity in C2 aligns with previous findings highlighting the critical role of cell cycle regulation during reprogramming [69,70].

During the transition from C4 to C2 and then to C0, in addition to the aforementioned cell cycle-related genes, other genes such as *Stat5a*, *Stat1*, *Src*, and *Jun*, which are closely associated with cell proliferation and differentiation, also show active regulatory involvement. This indicates that regulatory processes, such as the cell cycle and cell proliferation, become progressively more active during this transition (Fig. 4F). Moreover, the gene like *Trp53*, which is associated with apoptosis and DNA repair, also begins to exhibit regulatory activity during this transition (Fig. 4F). The processes of apoptosis and DNA repair are essential for cellular reprogramming, as they ensure the survival and proper differentiation of reprogrammed cells [71–73]. Next, the second transition point is identified at C0, from which 2 distinct trajectories emerge as C0 to C1 and C0 to C5 (Fig. 4F). Compared to C1, C5 has more regulatory links associated with maintaining genome stability, which involve regulators such as *Gnas*, *Ctnnb1*, *Trp53*, and *Hdac1* (Fig. 4F). These regulators contribute to a tight regulation network that ensures cellular homeostasis and stemness during reprogramming [74,75]. We summarized the entire dynamic process using GO enrichment analysis and calculated the Jaccard similarity between these cell types (clusters) (Fig. 4G and Methods). As expected, the similarity gradually decreased as the reprogramming process progressed.

Overall, the results derived from our SiCNet method demonstrate its effectiveness in uncovering the core regulatory mechanisms during the reprogramming process. Building on these findings, the newly identified regulators present potential targets for perturbation, enabling us to assess their impact on reprogramming efficiency.

### Identifying the developmental tendency and the dynamics of regulation in human hematopoiesis

**Fig. 5.**
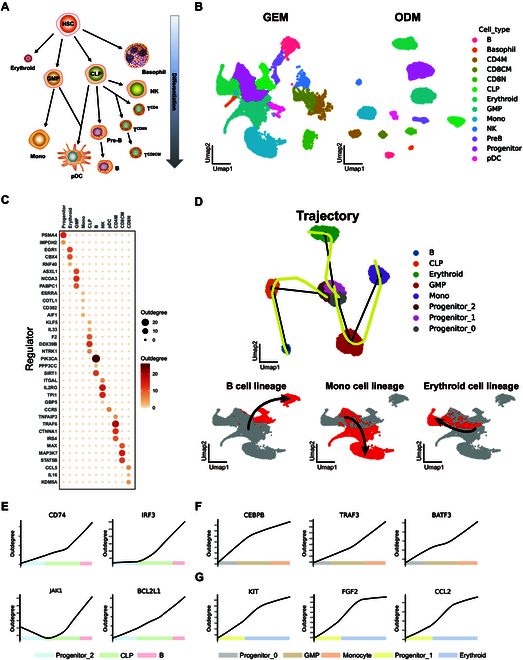
Identifying the developmental tendency of progenitor cells and discovering the dynamics of regulation in human hematopoiesis. (A) Hierarchical cartoon representation of the human hematopoietic cell types identified in scRNA-seq profiles of bone marrow mononuclear cells (BMMC) from healthy donors. pDC, plasmacytoid dendritic cell; Pre-B, pre-B cell; CLP, common lymphoid progenitor; GMP, granulocyte–monocyte progenitor; NK, natural killer cell; TCD4, CD4^+^ T cell; TCD8N, CD8^+^ naive T cell; TCD8CM, CD8^+^ central memory T cell; TCD4M, memory CD4^+^ T cells. (B) UMAP of the human hematopoietic cell types identified in GEM and ODM data. (C) Top regulator identified from each refined cell type-specific network based on target count. (D) Inferred potential development trajectories are divided into 3 lineages: B cell lineage, monocyte lineage, and erythroid cell lineage. (E) Examples of regulators with activated patterns of regulatory activity found in B cell lineage. The blue–green–red strip represents the development trajectories of progenitor–CLP–B lineage. (F) Examples of regulators with activated patterns of regulatory activity found in monocyte lineage. The gray–brown–orange strip represents the development trajectories of progenitor–GMP–Mono lineage. (G) Examples of regulators with activated patterns of regulatory activity found in erythroid cell lineage. The yellow–violet strip represents the development trajectories of progenitor–erythroid lineage.

To further demonstrate the utility of SiCNet in differential fields, we obtained scRNA-seq data of bone marrow mononuclear cells (BMMC) from healthy donors (Fig. 5A and B) [76,77]. We constructed the cell-specific network for each individual cell based on SiCNet, and then integrated and filtered the networks for each cell type to obtain the cell type-specific networks. Subsequently, we identified the top regulators exhibiting potential regulatory activity for each cell type (Fig. 5C). For example, *EGR1*, a gene known for its role in hematopoiesis, plays a critical role in the development and maturation of erythroid cells [78]. In addition, *PIK3CA*, a key regulator of the PI3K/AKT signaling pathway, is essential for B cell receptor (BCR) signaling, which is crucial for B cell development and function [79]. Furthermore, *TRAF6* contributes to the activation of nuclear factor-κB (NF-κB) and AP-1 transcription factors during CD4^+^ T cell development [80,81]. These findings indicate that the SiCNet method can accurately identify the specific regulatory genes in the differentiation and maturation of various immune cell types in human hematopoiesis.

Rather than focusing on the static regulation, we place more emphasis on the dynamic changes during the developmental process. By applying the SiCNet method, we can precisely model the dynamic regulations involved in hematopoietic development. This approach allows us to capture transient states and regulatory shifts, which provides a clearer and more comprehensive understanding of the underlying mechanisms. For hematopoietic progenitor cells, we consider that their developmental regulation may perform directional tendencies. We classified the progenitor cells into distinct subtypes and used Slingshot [68] to infer the developmental trajectories of these subtypes toward 3 lineages: B cells, monocytes, and erythroid cells (Fig. 5D). Using these cells along the lineage as a representation of developmental order, we can investigate the dynamic regulations of the development-associated regulators and uncover potential regulatory processes. For each regulator, its regulatory activity along the developmental lineage can be represented as a curve showing its changes from progenitor cells to a specific cell type. This curve is fitted based on the number of target genes regulated by the regulator in each cell along the lineage.

In the lineage of B cell development (Fig. 5E, Fig. S8, and Table S5), we found a series of increased activity of regulators. For example, *CD74*, a cell surface receptor for the cytokine macrophage migration inhibitory factor (MIF), plays a critical role in B cell development [82]. Our findings suggest that the number of target genes regulated by *CD74* progressively increases from progenitor cells to mature B cells. This observation is consistent with previous studies, which demonstrate that alterations in *CD74* expression significantly promote B cell differentiation and survival [83]. Given these characteristics, we believe that *CD74* may play a significant role in directing hematopoietic stem cells (HSCs) to differentiate into the B cell lineage by establishing new regulatory pathways. Similarly, *IRF3* is known to play central roles in cell differentiation and development, as well as in signaling downstream of various pathways, such as the Toll-like receptor (TLR) and BCR signaling pathways [84]. Importantly, BCR signaling is crucial for regulating B cell activation and survival, contributing to a balanced immune response [85]. The increase in *IRF3* regulation may integrate various signals from the BCR to promote the differentiation of naïve B cells into mature forms, such as plasma cells and memory B cells, which is essential for effective immune function [86]. In addition, *JAK1* is essential for maintaining the survival of mature B cells in peripheral tissues, ensuring effective immune responses and the generation of memory B cells. In the early stages of B cell development within the bone marrow, the increase of *JAK1* activation promotes the proliferation and differentiation of pro-B cells into pre-B cells by transducing signals from both the BCR and cytokines [87]. *BCL2L1*, also known as *BCL-XL*, plays a critical role in B cell development by protecting cells from programmed cell death. It helps promote the survival of B cells at various stages, particularly during the transitional and mature phases [88,89]. The increased activity of *BCL2L1* also promotes B cell maturation in a highly regulated manner [90].

In the lineage of monocyte cell development (Fig. 5F, Fig. S9, and Table S6), we identified several potential regulators that may be involved in promoting the survival and proliferation of monocytes during myeloid lineage differentiation. Specifically, *CEBPB* modulates the NF-κB signaling pathway to promote inflammatory responses, further facilitating the generation and functional maturation of monocytes [91–93]. Its increased regulation is required for the terminal differentiation and maturation of granulocytes and monocytes [94]. Moreover, the up-regulation of *TRAF3* is essential for monocyte activation, as it plays a pivotal role in cytokine signaling and cell survival, thereby facilitating the differentiation and immune function of monocytes [95,96]. In addition, we found that *BATF3* has a high activity during the development process, and it may play a pivotal role in driving the differentiation and functional maturation of monocytes. This suggests that *BATF3* may regulate critical genes involved in immune responses, contributing to the efficient transition of progenitor cells into fully developed monocytes. Furthermore, *BATF3* may influence the ability of these monocytes to mediate immune functions, such as antigen presentation and cytokine production.

In the lineage of erythroid cell development (Fig. 5G, Fig. S10, and Table S7), we found that regulators like *KIT*, *FGF2*, and *CCL2* exhibit up-regulated activity, suggesting their essential roles in promoting erythropoiesis. *KIT* is a receptor tyrosine kinase that plays a critical role in erythroid progenitor cell survival and differentiation. Binding its ligand, stem cell factor (SCF), *KIT* activates downstream signaling pathways such as the PI3K/AKT and MAPK/extracellular signal–regulated kinase (ERK) pathways. These pathways promote cell proliferation and prevent apoptosis during early erythroid development [97,98]. The increase of *KIT* activation is also proved to modulate terminal erythroid differentiation and survival [99]. *FGF2* is the prototype member of the fibroblast growth factor (FGF) family and interacts with its receptors (FGFRs) to mediate the receptor phosphorylation and the activation of signaling pathways, such as RAS/MAPK and PI3K/AKT pathways, which are involved in cell proliferation and differentiation [100]. In addition, it can enhance the formation and maintenance of blood cells [101,102]. Surprisingly, our findings suggest that *CCL2* may play a potential regulatory role in erythroid development. A previous study has shown that *CCL2* interacts with *ACKR1* to maintain an important physiological role in the erythroid lineage [103].

In summary, the SiCNet method effectively identifies known regulatory mechanisms while also uncovering potential regulatory insights within hematopoietic development. This capability enhances our challenge to explore both established and novel regulatory pathways, thereby advancing our knowledge of hematopoiesis and its associated mechanisms.

## Discussion

In this paper, we present SiCNet, a method for causal network inference, to construct cell-specific network and analyze dynamic regulations from single-cell transcriptome data. The SiCNet method integrates causal inference principles and prior knowledge-based networks to predict gene-level causal relationships in individual cells. Considering the variability in gene expression data and the robustness of gene regulation, we transform cell-specific networks into ODM for downstream dimension reduction and clustering analysis, which allows us to identify cell populations with greater accuracy than GEM. Importantly, we can use the SiCNet method to uncover regulatory insights at the network level in cancer progression, cellular reprogramming, and hematopoiesis. These regulations cannot be captured by current single-cell analysis, which focused solely on gene expression changes. In contrast, our SiCNet method measures the biological effects of each gene within the context of the cell-specific network. Furthermore, we capture dynamic regulatory changes at the single-cell level by focusing on individual cell differences rather than population-level trends. Notably, in addition to modeling at the single-cell level, our SiCNet method can also be applied to single samples to construct sample-specific networks. Moreover, SiCNet can be extended to spatial transcriptomics data, offering a powerful tool for capturing spatially dependent regulatory information that is crucial for understanding tissue architecture and function (Note S8).

GRN inference has long been a fundamental challenge in biology, with many unresolved issues, such as handling high-dimensional data with insufficient sample sizes, constructing networks from multiple samples, and addressing the complexities of time-series data, including temporal dependencies and feedback mechanisms. The SiCNet method incorporates causal inference while constructing networks for individual cells, offering a solution to some of these challenges. However, our approach does not account for nonlinearity, non-steady-state conditions, or mechanistic models, nor does it integrate other potentially informative data modalities, such as unspliced mRNAs, DNA methylation, chromatin accessibility, or cell lineage tracking. Despite these limitations, SiCNet assumes steady states and still uncovers rich biological information in non-steady-state developmental systems.

Single-cell technologies have revolutionized GRN inference and analysis, enabling comparisons at the level of individual cells across multiple modalities. Methods like SiCNet, which focus on single-cell-specific GRN inference, can reveal cellular heterogeneity and quantify gene regulation with higher throughput and resolution. This approach offers a broader perspective than individual gene expression analysis and paves the way for deeper biological discoveries and investigations.

## Methods

### Details of SiCNet algorithm

SiCNet is a data-driven and model-free algorithm for inferring gene-level causal relationships at the single-cell level (Fig. 1A). This method operates in 2 steps: (a) constructing a reference causal network from a designated reference dataset, and (b) inferring cell-specific causal networks for individual cells based on the reference network. The reference network serves as the benchmark to capture general causal relationships in gene regulation, while the cell-specific causal network utilizes the gene expression profile of an individual cell in combination with the reference network to infer causal relationships between genes.

#### Reference causal network construction

The first step is to infer the initial causal network, also referred to as the reference causal network, from a designated reference dataset (Fig. 1B). This dataset can be any single-cell gene expression data, but to make the results more meaningful, it is recommended to use data relevant to the research objectives. For example, in cancer research, it could correspond to normal adjacent tissue data, while in developmental studies, it might refer to progenitor cell-related data. The cell types within this set may consist of either a single-cell type or a mixture of multiple cell types. To enhance the interpretability of the inferred networks, we integrated the PPI network as prior knowledge into the inference process. We selected a gene $g^{r}$ and its first-order neighbor genes $g_{i}^{r}\in \left\{g_{1}^{r},g_{2}^{r},\ldots ,g_{n}^{r}\right\}$, as a unit (or subnetwork of $g^{r}$) (Fig. 1B). The r in $g^{r}$ refers to “reference”, indicating the gene expression profile of $g^{r}$ comes from the reference dataset. We assume that all these neighbors of gene $g^{r}$are the cause of $g^{r}$, implying that $g^{r}$ is regulated by these neighbor genes. Then, we inferred the causal relationship between $g^{r}$ and each $g_{i}^{r}$, respectively. In detail, we utilized a linear regression model to represent the relationship between $g^{r}$ and the presence or absence of $g_{i}^{r}$, as shown in Eqs. 1 and 2.

$$g^{r}=f_{1}\left(g_{i}^{r},Z^{r}\right)+\varepsilon_{1}^{r}=\sum_{j=1}^{n}\alpha_{j}g_{j}^{r}+\varepsilon_{1}^{r}$$ (1)

$$g^{r}=f_{2}\left(Z^{r}\right)+\varepsilon_{2}^{r}=\sum_{j=1,j\neq i}^{n}\beta_{j}g_{j}^{r}+\varepsilon_{2}^{r}$$ (2)

Here, the functions $f_{1}\left(g_{i}^{r},Z^{r}\right)$ and $f_{2}\left(Z^{r}\right)$ model the relationships between $g^{r}$ and its neighbors, with the key difference being whether $g_{i}^{r}$ is present or not. Accordingly, $Z^{r}$ represents the set of all first-order neighbor genes of $g^{r}$, excluding $g_{i}^{r}$. The parameters $\alpha_{j}$ and $\beta_{j}$ denote the regression coefficients corresponding to Eqs. 1 and 2, respectively. The residuals $\varepsilon_{1}^{r}$ and $\varepsilon_{2}^{r}$ represent the RMSE of the linear model corresponding to Eqs. 1 and 2, respectively. Notably, to ensure more reliable results, we applied 5-fold cross-validation on the samples (cells). Particularly, the dataset was randomly split into 5 approximately equal-sized subsets. In each iteration, one subset was used as the validation set, while the remaining 4 subsets were used as the training set. The linear regression model was trained on the training set and tested on the validation set to compute the loss values. This process was repeated for all 5 subsets, and the losses for each model were averaged across the 5 iterations to obtain the final loss values, which were referred to as $\varepsilon_{1}^{r}$ and $\varepsilon_{2}^{r}$.

The causal relationship between $g_{i}^{r}$ and $g^{r}$ can be quantified by a CSI $\omega_{r}\left(g_{i}^{r},g^{r}\right)$, which is described in Eq. 3.

$$\omega_{r}\left(g_{i}^{r},g^{r}\right)=\text{ln}\left(\frac{\varepsilon_{2}^{r}}{\varepsilon_{1}^{r}}\right)\;$$ (3)

The positive $\omega_{r}$ indicates the existence of a causal relationship from the gene $g_{i}^{r}$ to the gene $g^{r}$; otherwise, the absence of this causal relationship is inferred. Thus, by determining the sign of the CSI for each $g_{i}^{r}$ to $g^{r}$, we construct a reference causal network that serves as the benchmark for predicting cell-specific causal networks in the next step.

#### Cell-specific causal network inference

The next step is to infer cell-specific causal networks for individual cells based on the reference causal network (Fig. 1C). Similarly, we selected a gene $g^{c}$ and its first-order cause (regulatory) genes $g_{i}^{c}\in \left\{g_{1}^{c},g_{2}^{c},\ldots ,g_{m}^{c}\right\}$ from the reference causal network as a unit (or subnetwork of $g^{c}$). The c in $g^{c}$ refers to “specific cell”, indicating that the gene expression profile of $g^{c}$ comes from the particular cell. We used a set of gene expression of $g_{i}^{c}$ to estimate the expression of $g^{c}$ in each individual cell, as shown in Eq. 4. Then, we estimated the expression of $g^{c}$ when the corresponding cause (regulatory) gene $g_{i}^{c}$ was absent, as shown in Eq. 5.

$$g^{c}=f_{1}\left(g_{i}^{c},Z^{c}\right)+\varepsilon_{1}^{c}=\sum_{j=1}^{n}\alpha_{j}^{\prime }g_{j}^{c}+\varepsilon_{1}^{c}\;$$ (4)

$$g^{c}=f_{2}\left(Z^{c}\right)+\varepsilon_{2}^{c}=\sum_{j=1,j\neq i}^{n}\beta_{j}^{\prime }g_{j}^{c}+\varepsilon_{2}^{c}$$ (5)

Here, $f_{1}\left(g_{i}^{c},Z^{c}\right)\;$ and $f_{2}\left(Z^{c}\right)$ represent the estimated functions derived from the reference causal network. Accordingly, $Z^{c}$ represents the set of all first-order cause (regulatory) genes of $g^{c}$, excluding $g_{i}^{c}$. The parameters $\alpha_{j}^{\prime }$ and $\beta_{j}^{\prime }$ denote the regression coefficients corresponding to Eqs. 4 and 5, respectively. The residuals $\varepsilon_{1}^{c}$ and $\varepsilon_{2}^{c}$ denote the RMSE between the actual expression of $g^{c}$ and its corresponding predicted values $f_{1}\left(g_{i}^{c},Z^{c}\right)$ and $f_{2}\left(Z^{c}\right)$, respectively.

Similarly, the causal relationship between $g_{i}^{c}$ and $g^{c}$ can be determined by the sign of $\omega_{c}\left(g_{i}^{c},g^{c}\right)$ within the single cell, as shown in Eq. 6. If the value of $\omega_{c}$ exceeds zero, it indicates the existence of a causal influence of $g_{i}^{c}$ on $g^{c}$; otherwise, no such causal relationship exists.

$$\omega_{c}\left(g_{i}^{c},g^{c}\right)=\ln\left(\frac{\varepsilon_{2}^{c}}{\varepsilon_{1}^{c}}\right)\;$$ (6)

By performing causal predictions for each gene pair ($g_{i}^{c}$ to $g^{c}$) in every individual cell, we construct cell-specific causal networks.

### Background network

To obtain a background network as the basis for initial causal network construction, we downloaded the PPI network from the STRING database version 12.0 [104] (https://string-db.org/) and filtered out the edges with associated scores below 700.

### Network binarization

To simplify the calculation and subsequent analyses, we binarized both the reference causal network and all cell-specific networks. Thus, for the edge of gene $g_{i}$ to gene $g_{j}$ (i≠j) in the network, we have:

edgegi→gj=0,ω≤01,ω>0 (7)

### Network ODM from SiCNet method

Cell-specific networks constructed by SiCNet can be applied to various biological studies at the network level. However, the number of features described in a network in most scRNA-seq analyses is quite large. For instance, there are at least 100,000 gene pairs or direct edges supported by the STRING PPI network. In this paper, we transformed the cell-specific network from SiCNet method into a network ODM to capture network features while simultaneously reducing dimensionality, although SiCNets can also be directly used or reduced through other methods for clustering analysis. Thus, for gene $g_{i}$ in the network of cell *k*, we have:

$${\mathrm{ODM}}_{g_{i}}^{k}=\sum_{i=1,i\neq j}^{m}{\text{edge}}_{g_{i}\to g_{j}}$$ (8)

Then, we can get a matrix ODM with m×n elements, where 𝑚 represents the number of genes and 𝑛 represents the number of cells. $g_{i}$ denotes the regulator in the network, and $g_{j}$ represents the regulated gene (target gene). In the ODM, the count of $g_{j}$ is considered as the outdegree of $g_{i}$. We use the NetworkX Python package to identify the outdegree of the network in each cell.

The ODM retains the same dimensions as the original GEM, with the same number of rows and columns, but it represents the higher-order information of each gene within the network rather than the gene expression levels. This matrix can be seamlessly analyzed using any standard scRNA-seq algorithm for cell clustering, dimensionality reduction, and pseudo-trajectory analysis by simply substituting the original GEM with our ODM. Thus, our SiCNet method introduces a novel approach for analyzing scRNA-seq data at the network level.

### Benchmark datasets

In this work, we collected 5 single-cell datasets to demonstrate the advantages of our method in cell clustering. The cell type annotations were derived from the original publications, where they were determined by the authors using their biological expertise. Detailed descriptions and sources of all datasets are provided below.

The Biase dataset contains 49 cells with 3 types: 2-cell mouse embryos, 4-cell mouse embryos, and zygotes [105].

Enge dataset contains pancreatic cells from 8 human volunteers, comprising a total of 2,282 cells across 6 distinct cell types [106].

Darmanis dataset is a human brain dataset characterized by its complexity, comprising 7 different cell types and 331 cells [107].

Fink dataset is a total of 3,045 single-cell transcriptomes of stromal cells from human adult ureters, encompassing 7 distinct cell types [108].

Goolam dataset consists of sequencing results from mouse embryo cells including 5 cell types and 124 cells [109].

All these datasets can be obtained at the Data Availability section.

### Student’s *t* test

The “scipy.stats.ttest_ind” function in Python was employed to implement Student’s *t* test for the different outdegrees or gene expression between 2 groups, such as the healthy adjacent region and the core region in Fig. 3. The *P* values were adjusted for multiple testing using the FDR method.

### Chi-square test

The “scipy.stats.chi2_contingency” function in Python was employed to implement chi-square test on the rate of activity change for each edge from the healthy adjacent region to the core region in Fig. 3, with *P* values adjusted for multiple testing using FDR.

### Evaluation metrics

ARI [110], AMI [111], NMI [112], and FMI [113] are used as metrics to evaluate the clustering performance.

The ARI measures the similarity between 2 cluster sets, adjusting for the chance grouping of elements. It considers all pairs of samples and counts pairs that are assigned in the same or different clusters in the predicted and true clusters. The ARI is defined as:

$$\mathrm{ARI}=\frac{\sum_{\mathrm{ij}}\left(\begin{array}{c} n_{\mathrm{ij}}\\ 2 \end{array}\right)-\frac{\left[\sum_{i}\left(\begin{array}{c} a_{i}\\ 2 \end{array}\right)\sum_{j}\left(\begin{array}{c} b_{j}\\ 2 \end{array}\right)\right]}{\left(\begin{array}{c} n\\ 2 \end{array}\right)}}{0.5\left[\sum_{i}\left(\begin{array}{c} a_{i}\\ 2 \end{array}\right)+\sum_{j}\left(\begin{array}{c} b_{j}\\ 2 \end{array}\right)\right]-\frac{\left[\sum_{i}\left(\begin{array}{c} a_{i}\\ 2 \end{array}\right)\sum_{j}\left(\begin{array}{c} b_{j}\\ 2 \end{array}\right)\right]}{\left(\begin{array}{c} n\\ 2 \end{array}\right)}}$$ (9)

where n is the total number of samples, $n_{\mathrm{ij}}$ is the number of samples in both cluster i and cluster j, and $a_{i}$ and $b_{j}$ are the sum of elements in cluster i of the true clustering and cluster j of the predicted clustering, respectively.

AMI adjusts the mutual information score for chance, providing a normalized measure of similarity between 2 cluster sets. It considers how much information is shared between the 2 cluster sets, accounting for the expected mutual information between 2 random cluster sets. The AMI is defined as:

$$\mathrm{AMI}=\frac{\mathrm{MI}\left(U,V\right)-E\left[\mathrm{MI}\left(U,V\right)\right]}{\max\left(H\left(U\right),H\left(V\right)\right)-E\left[\mathrm{MI}\left(U,V\right)\right]}$$ (10)

where $\mathrm{MI}\left(U,V\right)$ is the mutual information between the true labels U and predicted labels V, $H\left(U\right)$ and $H\left(V\right)$ are the entropies of U and V, and $E\left[\mathrm{MI}\left(U,V\right)\right]$ is the expected mutual information.

NMI measures the similarity between 2 cluster sets, normalized to scale between 0 (no mutual information) and 1 (perfect correlation). It is defined as:

$$\mathrm{NMI}=\frac{\mathrm{MI}\left(U,V\right)}{\sqrt{H\left(U\right)\cdot H\left(V\right)}}$$ (11)

where $\mathrm{MI}\left(U,V\right)$ is the mutual information between the true labels U and predicted labels V, and $H\left(U\right)$ and $H\left(V\right)$ are the entropies of U and V.

The FMI is a geometric mean of precision and recall used for evaluating the quality of clustering by comparing the pairwise similarity between the true and predicted clusters. It is defined as:

$$\mathrm{FMI}=\sqrt{\frac{\mathrm{TP}}{\mathrm{TP}+\mathrm{FP}}\cdot \frac{\mathrm{TP}}{\mathrm{TP}+\mathrm{FN}}}$$ (12)

where TP (true positive) is the number of pairs correctly clustered together, FP (false positive) is the number of pairs incorrectly clustered together, and FN (false negative) is the number of pairs incorrectly not clustered together.

## Data Availability

The benchmark datasets in this work were collected from the NCBI GEO (https://www.ncbi.nlm.nih.gov/geo/) database with accession numbers GSE57249, GSE81547, GSE67835 and GSE184111 and the ArrayExpress database, hosted by EMBL-EBI (https://www.ebi.ac.uk/), with accession number E-MTAB-3321. The single-cell RNA-Seq data for colorectal cancer were obtained from GEO with accession number GSE144735. The mouse cellular reprogramming scRNA-seq data were accessed through GEO with accession number GSE108222. The human blood scRNA-seq data were available through GEO with accession number GSE139369. The source code of the SiCNet algorithm is available at https://github.com/Huang-XZ-Sandy/SiCNet.

## Supplementary Materials

Supplementary 1 Supplementary Notes S1 to S10 Figs. S1 to S24 Tables S1 to S8
