# Supplementary material for: Constructing Cell-Specific Causal Networks of Individual Cells for Depicting Dynamical Biological Processes
Source: Research (Wash D C). 2025 Jun 27;8:0743. doi: 10.34133/research.0743 (PMC12202884; doi:10.34133/research.0743)
Supplement: Supplementary 1 — Supplementary Notes S1 to S10 Figs. S1 to S24 Tables S1 to S8 [file research.0743.f1.pdf]

SiCNet: constructing cell-specific causal networks of individual cells for depicting dynamical biological processes

Supplementary Notes

Supplementary Note 1. The details of analyzing colorectal cancer progression patterns

We downloaded TPM-normalized single-cell RNA-seq data of human colorectal cancer (see `Data availability`). The dataset includes 27,414 cells from 6 colorectal cancer patients (KUL01, KUL19, KUL21, KUL28, KUL30 and KUL31), and covers the border and core regions of tumors as well as matched normal mucosa. To investigate the molecular mechanisms, we selected cell types representing epithelial cells ($EPCAM^{+}$) from different tumor regions (border and core) and the adjacent healthy tissue. Using the SiCNet method, we counted the outdegrees of each regulator and compared them across the three different regions using the two-sided Student's *t*-test in Python. The *p*-values were adjusted for multiple testing using the False Discovery Rate (FDR) method. Meanwhile, we identified up-regulated edges representing regulator-target gene interactions across the three regions. We then performed a chi-square test on the rate of activity change for each edge in Python, with p-values adjusted for multiple testing using FDR.

Supplementary Note 2. The details of calculating dynamic network biomarker (DNB) scores for 6 colorectal cancer samples

To characterize each sample, we first integrated the cell-specific networks from each region, converting the binary edges (presence or absence indicated by 1 or 0) into edge weights that represent the proportion of cells sharing each connection across the combined networks. We then applied a threshold, retaining only those edges with cellular proportions equal to or greater than 0.7. This resulted in a final, cell state-specific network for each individual sample. Correspondingly, the single-cell expression data were aggregated by averaging the expression values of cells within each sample, resulting in an average gene expression vector for each sample. For each region, we obtained the average gene expression data within 6 samples. Subsequently, the *l*-DNB method [1] was employed to compute the dynamic network biomarker (DNB) scores for genes within each sample's network. The genes were then ranked according to their DNB scores, and the top 50 genes were selected to constitute the DNB module for each sample. The global DNB score for each sample was calculated by summing the DNB scores of these top 50 genes.

Supplementary Note 3. The details of analyzing mouse cellular reprogramming patterns

We downloaded mouse cellular reprogramming scRNA-seq datasets (see `Data availability`) for the 6 time points (MEF, D3, D6, D9, D12 and mESC) and concatenated the expression data from two replicates at each time point and normalized the combined matrix for sequencing depth and variance stabilization using a simplified implementation of the default Scanpy pipeline [2]. After applying our SiCNet method, we generated ODMs for each time point and concatenated them across all time points to produce our final ODM, containing 10,807 genes and 3,152 cells (one of the MEF

replicates were used as the reference cell set). We then applied the Leiden clustering method with default parameters to determine the optimal clustering of the final ODM, based on the clustering from the MEF to the mESC timepoints. We used Slingshot [3] to infer the pseudotime trajectory of the reprogramming pattern. We constructed cell cluster-specific networks with a cellular proportion threshold of ⩾ 0.7 in each cluster and visualized the networks using Cytoscape [4]. We calculated the Jaccard similarity between the refined networks in Python.

Supplementary Note 4. The details of analyzing human hematopoietic differential patterns

We obtained the human hematopoietic differentiation scRNA-Seq data (see `Data availability`) of bone marrow mononuclear cells from healthy donors [5]. We used the annotated cell clusters and cell differential lineage in reference [6] for the scRNA-seq data. We divided the data into separate expression matrices for each annotated cell type and preprocessed them using the default Scanpy pipeline. Then, we used our SiCNet method to construct networks for individual cells. We also used Slingshot [3] to infer the refined differential trajectory of the human blood developmental patterns across three classical lineages: progenitor cells differentiating into B cells, monocytes and erythroid cells.

Supplementary Note 5. Regulatory network analysis for the B cell lineage

Cell type-specific GRNs construction was also performed across the B-cell trajectory in order to illustrate cell fate determining regulators and their putative connections with prominent gene markers across B cell development (Supplementary Fig. 11-13). Similar to the previous case, we constructed cell type-specific networks with a cellular proportion threshold of ⩾ 0.7. During the whole lineage, we found that the edges like CCNA2-ATM, BLM-TOP3A and FBXO5-CDC20 are active. These regulations activate the cell cycle pathway, thereby facilitating the progression of B cell development and ensuring the proper maturation and differentiation of B cells within the immune system [7-9].

Supplementary Note 6. Regulatory network analysis for the Monocyte cell lineage

Following the trajectory towards Monocyte cell development, we also identified the aforementioned regulators and regulatory connections underlying HSC and Progenitors program. Regulators like *HSP90AB1*, *PTGS2* and *JUND* were involved in IL-17 signaling pathway and TNF signaling pathway, contributing to the development and function of monocytes by enhancing inflammatory responses, cell migration, and marker gene expression [10, 11]. In addition, we constructed the cell type-specific GRNs with a cellular proportion threshold of ⩾ 0.7 and found that the regulatory activities of regulators such as *EIF3I*, *SNRPD3* and *HSP90AB1* became increasingly active, as reflected in the growing density of the network (Supplementary Fig. 14-16). It indicted that these regulators may play an important role in monocyte development.

Supplementary Note 7. Regulatory network analysis for the Erythroid cell lineage

Following the trajectory towards Erythroid cell development, we found that regulators like *PRKAA1*,

NDUFA13 and *ALOX15* were involved in cellular response to oxygen-containing compound, suggesting their potential roles in modulating erythropoiesis. These regulators may contribute to the adaptation of erythroid cells to changes in oxygen levels, which is crucial for maintaining proper red blood cell function and ensuring efficient oxygen transport [12]. Additionally, we also constructed the cell type-specific GRNs with a cellular proportion threshold of ⩾ 0.7 and found that the regulatory activities of regulators such as *IRF1*, *IRF9* and *PTPRC* became increasingly active, as reflected in the growing density of the network (Supplementary Fig. 17-18). It indicted that these regulators may play an important role in erythroid development.

Supplementary Note 8. Analysis of spatially dependent regulatory networks in the mouse olfactory bulb

We obtained spatial transcriptomic data of the mouse olfactory bulb from the reference [13] and used the 12th replicate slice for spatial transcriptomic analysis (Supplementary Fig. 19a). We applied SiCNet to infer spot-specific causal networks. Subsequently, we performed clustering of the spots using Leiden algorithm and identified five distinct clusters. Based on prior literature [13], we annotated these clusters as the granular cell layer (GCL), outer plexiform layer (OPL), olfactory nerve layer (ONL), mitral cell layer (MCL), and glomerular layer (GL) (Supplementary Fig. 19b). For each cluster, we integrated the spot-specific causal networks and constructed cluster-specific causal network. The cluster-specific causal network was integrated based on the criterion that the proportion of spots exhibiting a particular edge should be greater than or equal to 70% within the cluster and was visualized by using Cytoscape [4]. We found that among the top 10 high regulatory activity regulators in each cluster, *Apoe*, a lipid transporting protein and widely expressed in the olfactory pathway [14], was predominantly present in GL, GCL and MCL (Supplementary Fig. 20-22). Previous studies have shown that *Apoe* is a unique and early biomarker in the olfactory nerve and is important for neuroregeneration of the rodent olfactory system [15, 16]. Our prediction indicates that *Apoe* may modulate neuronal activity within these layers, potentially contributing to tissue repair and recovery in the olfactory system, particularly following injury or degeneration. In addition, *Gsk3b* and *Snap25* have high regulatory activity in ONL (Supplementary Fig. 23). *Gsk3b* is known to be involved in the regulation of the synaptic plasticity [17], while *Snap25* has been identified as a predictor of neuronal loss or impaired synaptogenesis when its expression decreases [18]. Our prediction suggests that the regulatory activity of these regulators is localized to specific regions within the olfactory system, particularly the ONL, highlighting their potential spatial influence in synaptic regulation.

Supplementary Note 9. Benchmark of cell clustering methods

To illustrate the effectiveness of the ODM generated by SiCNet in cell clustering, we compared it with the original GEM using five commonly used single-cell clustering methods. Notably, our focus is not on the clustering methods themselves, but on comparing the GEM with our ODM. To ensure a fair comparison, the same parameters were applied across all methods for both GEM and ODM.

SC3 [19] uses a consensus clustering approach to achieve accurate and robust clustering of single-cell RNA-seq data. The process begins by filtering genes to reduce dimensionality, followed by

constructing distance matrices based on multiple metrics such as Euclidean, Pearson, and Spearman distances. Spectral transformations are applied to these matrices, and k-means clustering is performed on the transformed data to obtain an initial set of clusters. This process is repeated across various parameter combinations to create multiple clustering solutions. These solutions are then combined into a consensus matrix that captures the frequency with which each pair of cells is clustered together. Finally, hierarchical clustering is applied to the consensus matrix to produce the final clustering result, which is both stable and robust across different datasets.

CIDR [20] employs a novel imputation approach to estimate the gene expression values affected by dropouts, followed by principal coordinate analysis (PCoA) to reduce dimensionality. It constructs a dissimilarity matrix based on these imputed values and applies hierarchical clustering to the first few principal coordinates.

Scanpy [2] is a comprehensive toolkit for single-cell RNA-seq data analysis that includes a highly efficient clustering workflow. The data is reduced in dimensionality using principal component analysis (PCA). A mutual nearest neighbors (MNN) graph is constructed based on the PCA-reduced data to account for batch effects and align cells across batches. This MNN graph forms the basis for clustering using the Louvain or Leiden community detection algorithms, which group cells into clusters representing distinct cell types or states. The clusters are visualized using t-SNE or UMAP for easier interpretation and biological insight, allowing researchers to explore cellular heterogeneity and identify meaningful patterns within the dataset.

SIMLR [21] is a framework designed for clustering, dimension reduction, and visualization of single-cell RNA-seq data. SIMLR learns a cell-to-cell similarity matrix by integrating multiple kernels to capture various measures of distance between cells. This similarity matrix is then used to perform dimension reduction through a modified t-SNE approach and to identify cell clusters using affinity propagation or k-means clustering. SIMLR addresses the high dropout rates in single-cell data by employing a rank constraint in the similarity matrix and utilizing a diffusion-based method to enhance similarity measures, thereby improving clustering accuracy and visual representation.

Supercell [22] is a computational framework designed to simplify the analysis of large single-cell RNA-seq (scRNA-seq) datasets by merging highly similar cells into metacells. The method begins by constructing a k-nearest neighbor (kNN) graph based on transcriptomic similarity among cells. Metacells are formed by clustering densely connected cells using the walktrap algorithm, allowing users to define the number of metacells. This approach reduces data size, noise from dropout events, and computational requirements while preserving the global structure of the data.

Supplementary Note 10. The network size for real datasets

The reference network for the human colorectal cancer scRNA-seq data contains 17,056 genes and 129,465 edges. For each cell, we observed an average of 65,069 edges.
The reference network for the cellular reprogramming data contains 18,166 genes and 117,726 edges. For each cell, we observed an average of 59,132 edges.
The reference network for the human hematopoietic differentiation data contains 17,597 genes and 140,256 edges. For each cell, we observed an average of 63,645 edges.

Supplementary Figures

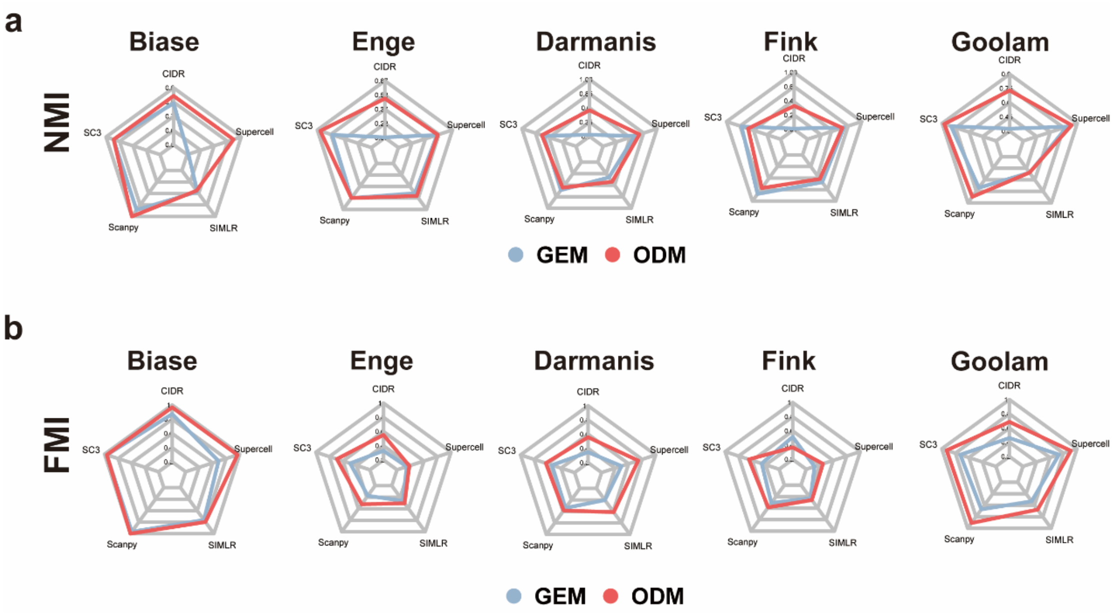

Supplementary Fig. 1. Five benchmark scRNA-Seq datasets demonstrated superior clustering performance with ODM compared to GEM over existing methods. a. The NMI metrics are used to measure performance of clustering. Clustering methods SC3, CIDR, Scanpy, SIMLR and Supercell are used to cluster datasets. b. The FMI metrics are used to measure performance of clustering. Clustering methods SC3, CIDR, Scanpy, SIMLR and Supercell are used to cluster datasets. GEM, gene expression matrix; ODM, network outdegree matrix; NMI, Normalized Mutual Information; FMI, Fowlkes-Mallows Index.

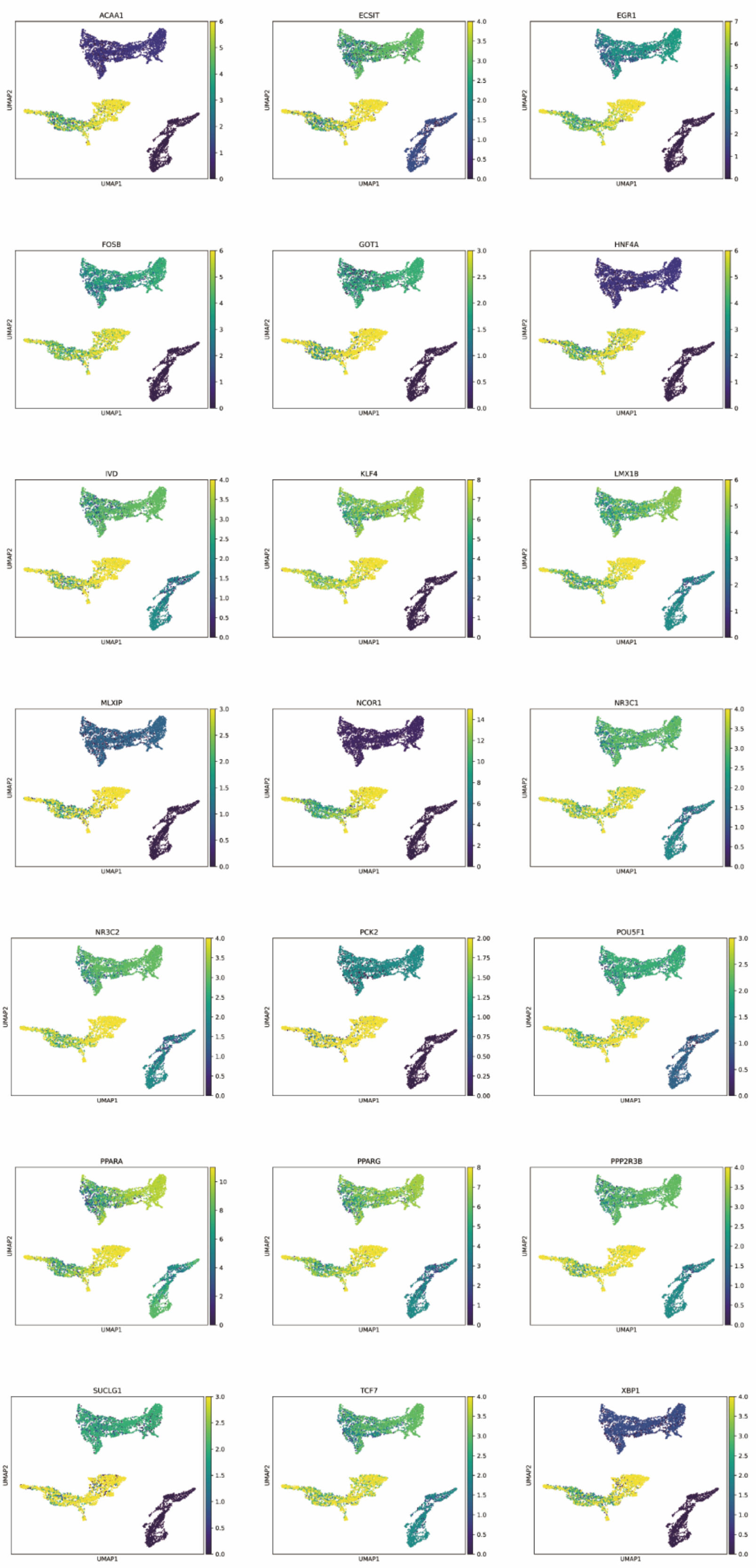

Supplementary Fig. 2. SiCNet reveals colorectal cancer progression regulatory patterns. UMAP visualization of ODM showing other key regulators with high regulatory activity across the gradient from the healthy adjacent region to the core region.

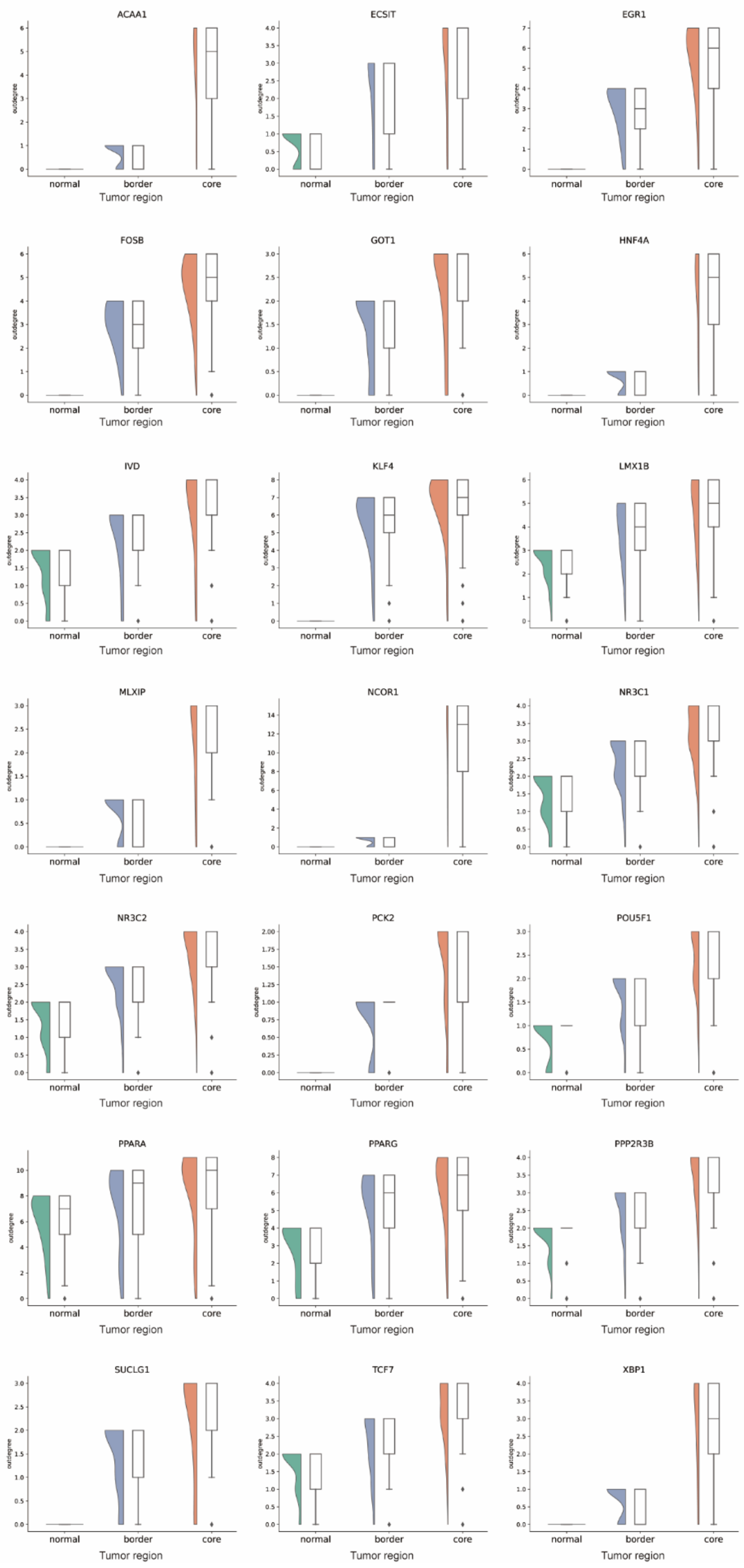

Supplementary Fig. 3. SiCNet reveals colorectal cancer progression regulatory patterns. The raincloud plot of other key regulators' regulatory activity from the healthy adjacent region to the core region.

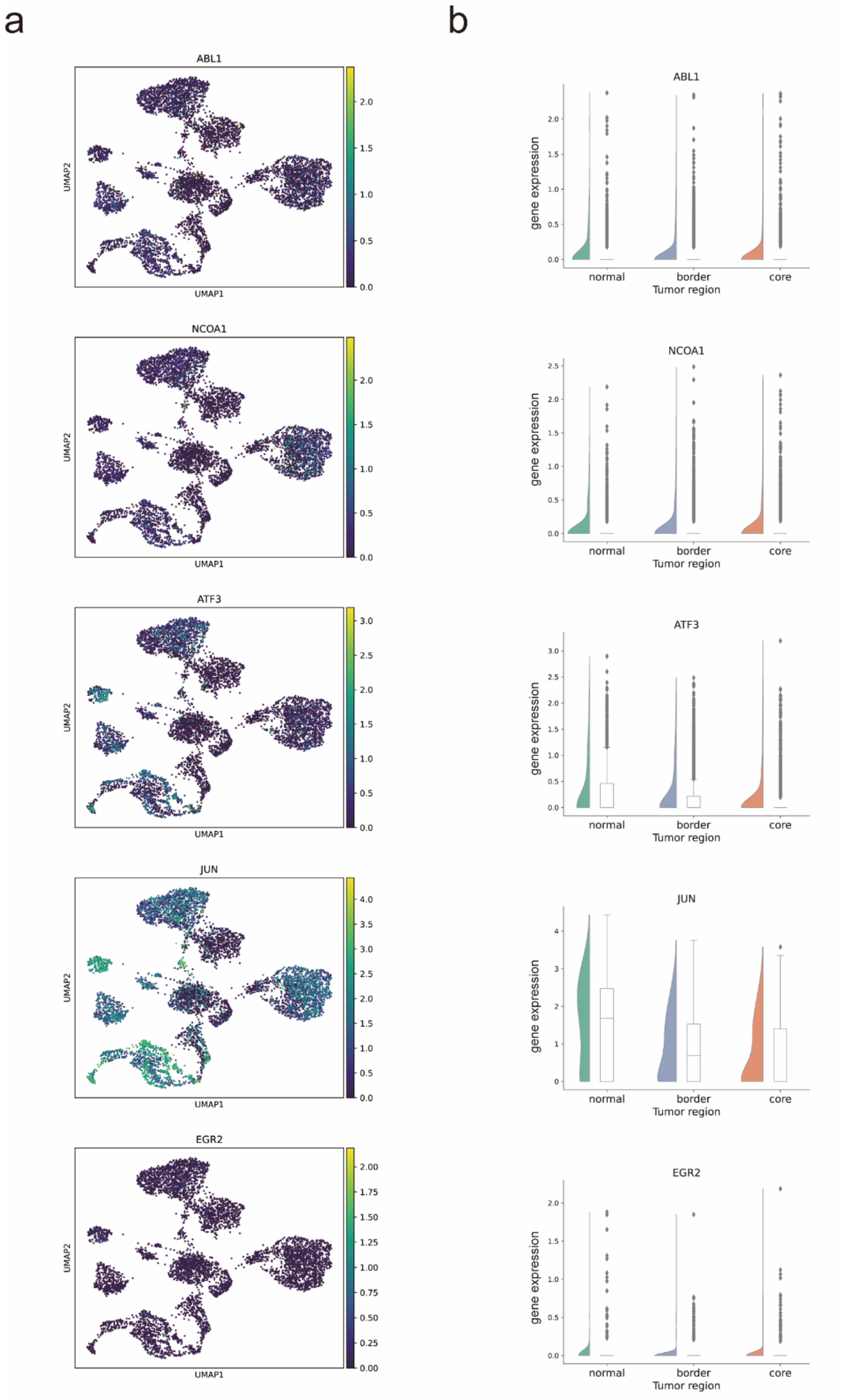

Supplementary Fig. 4. SiCNet reveals colorectal cancer progression regulatory patterns. a. UMAP visualization of GEM showing key regulators identified by SiCNet method across the gradient from the healthy adjacent region to the core region. b. Corresponding raincloud plot of the key regulators' gene expression from the healthy adjacent region to the core region.

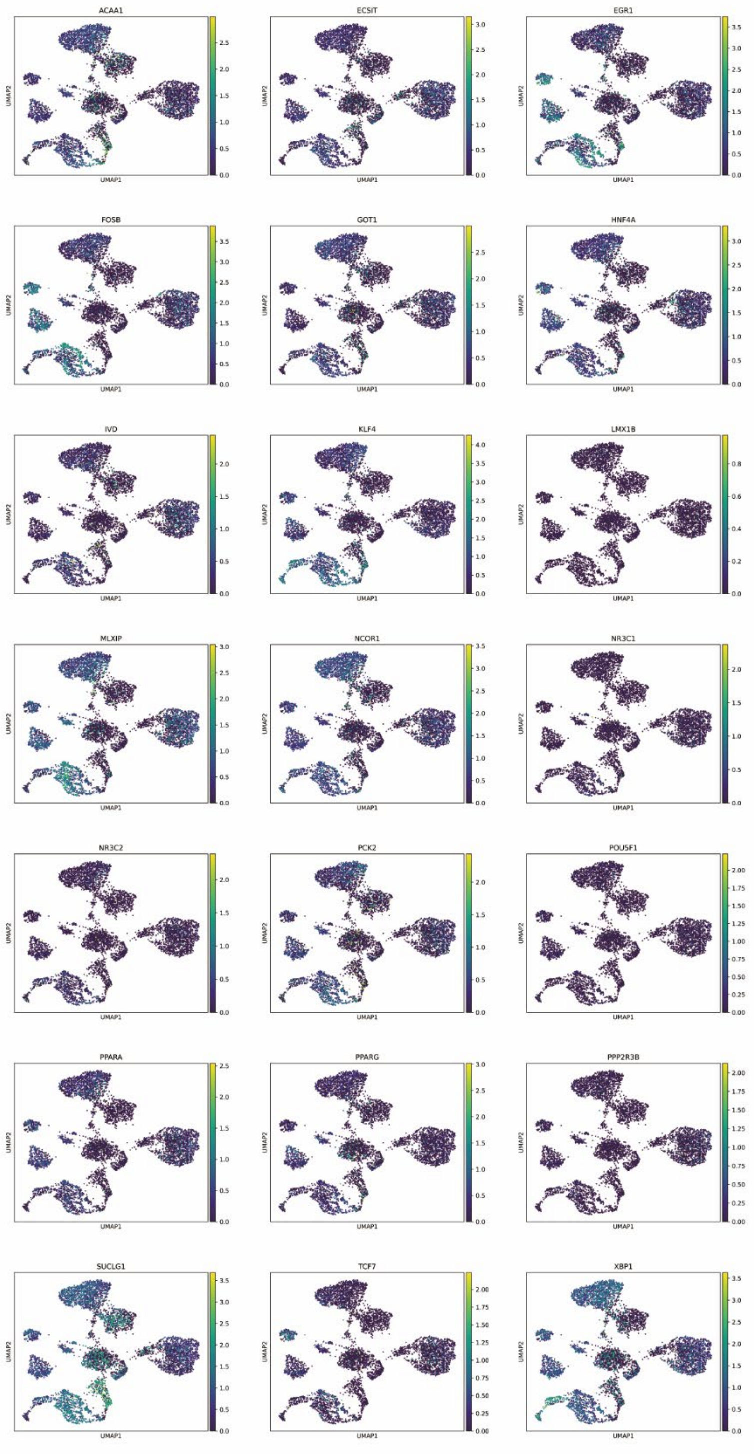

Supplementary Fig. 5. SiCNet reveals colorectal cancer progression regulatory patterns. UMAP visualization of GEM showing other key regulators identified by SiCNet across the gradient from the healthy adjacent region to the core region.

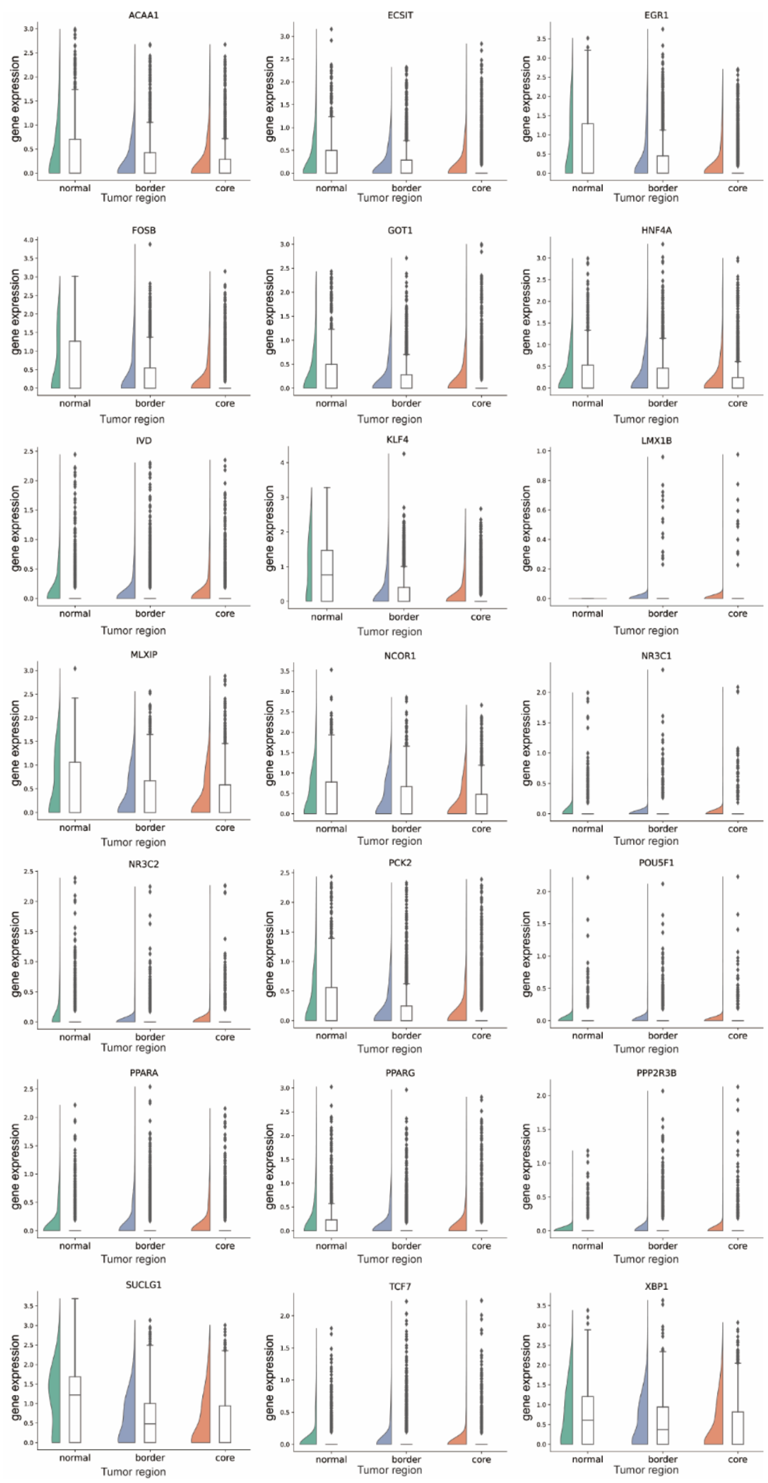

Supplementary Fig. 6. SiCNet reveals colorectal cancer progression regulatory patterns. The raincloud plot of other key regulators' gene expression from the healthy adjacent region to the core region.

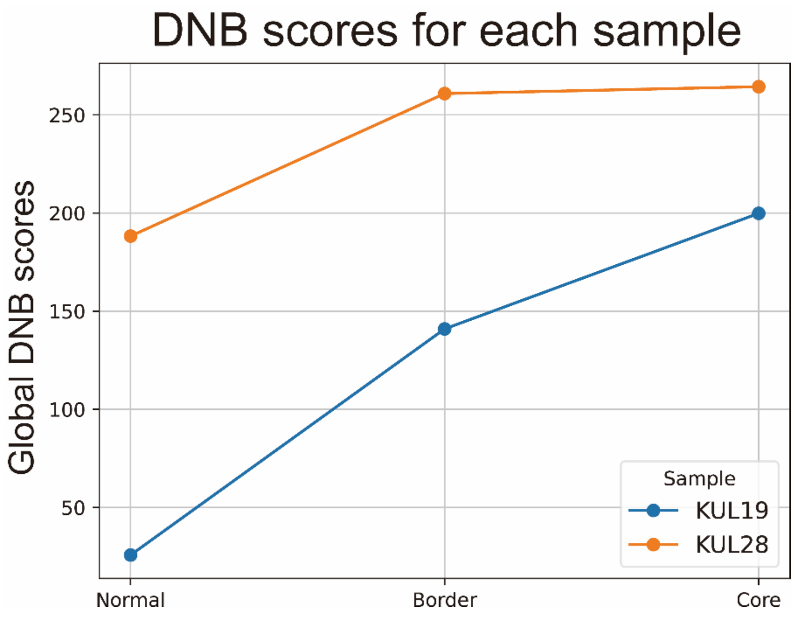

Supplementary Fig. 7. Identification of critical states for cancer progression for each sample.

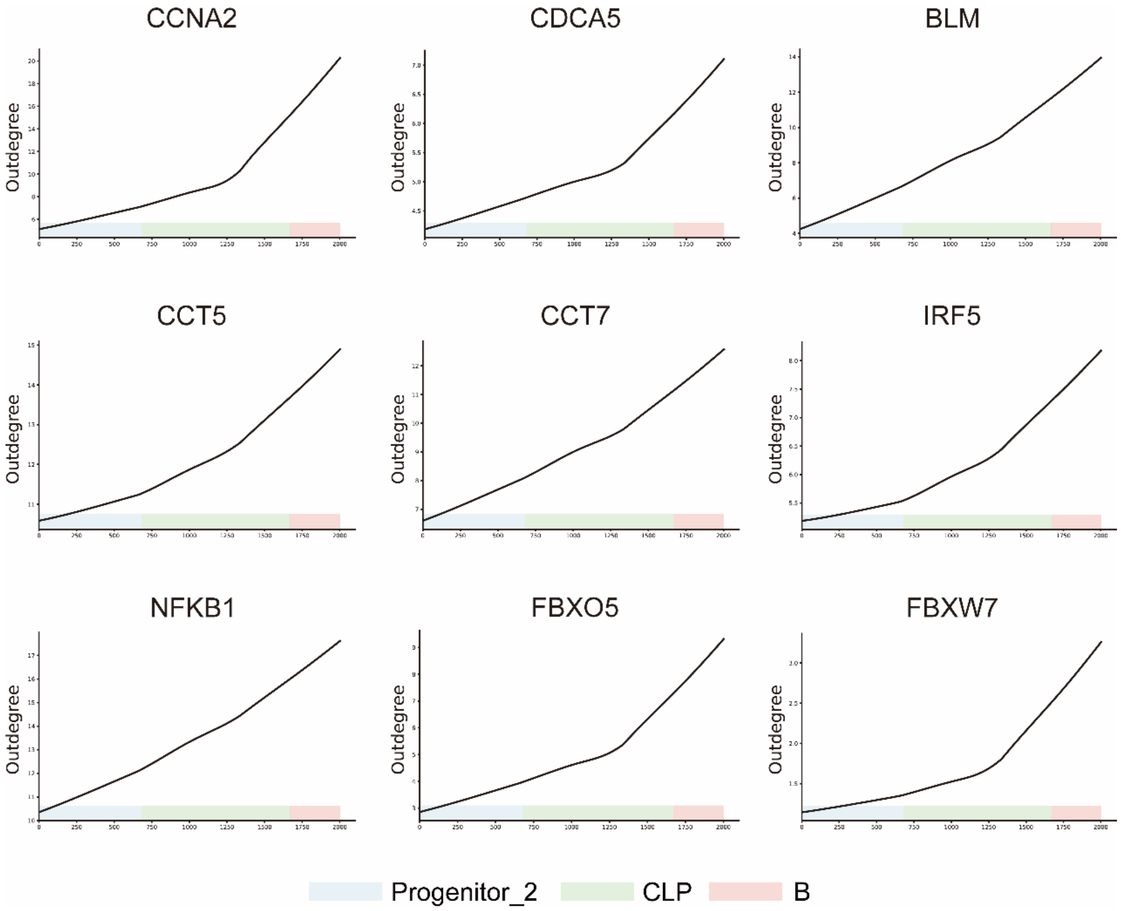

Supplementary Fig. 8. The identified regulators of dynamic changes in B cell lineage. Examples of regulators with activated patterns of regulatory activity found in B cell lineage. The blue-green-red strip represents the development trajectories of Progenitor-CLP-B lineage.

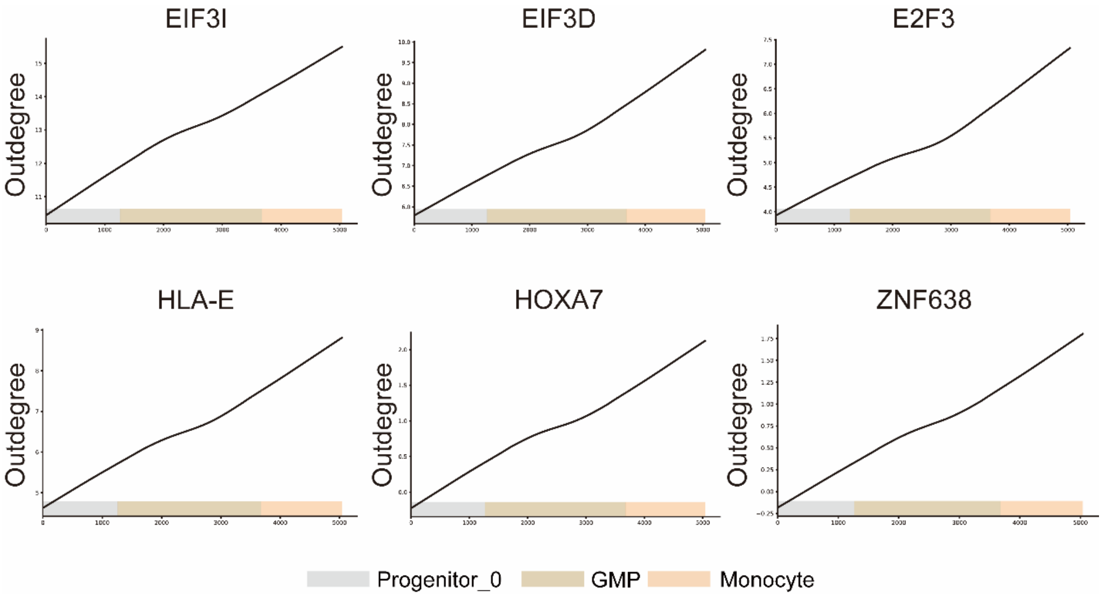

Supplementary Fig. 9. The identified regulators of dynamic changes in Monocyte cell lineage. Examples of regulators with activated patterns of regulatory activity found in monocyte lineage. The grey-brown-orange strip represents the development trajectories of Progenitor-GMP-Mono lineage.

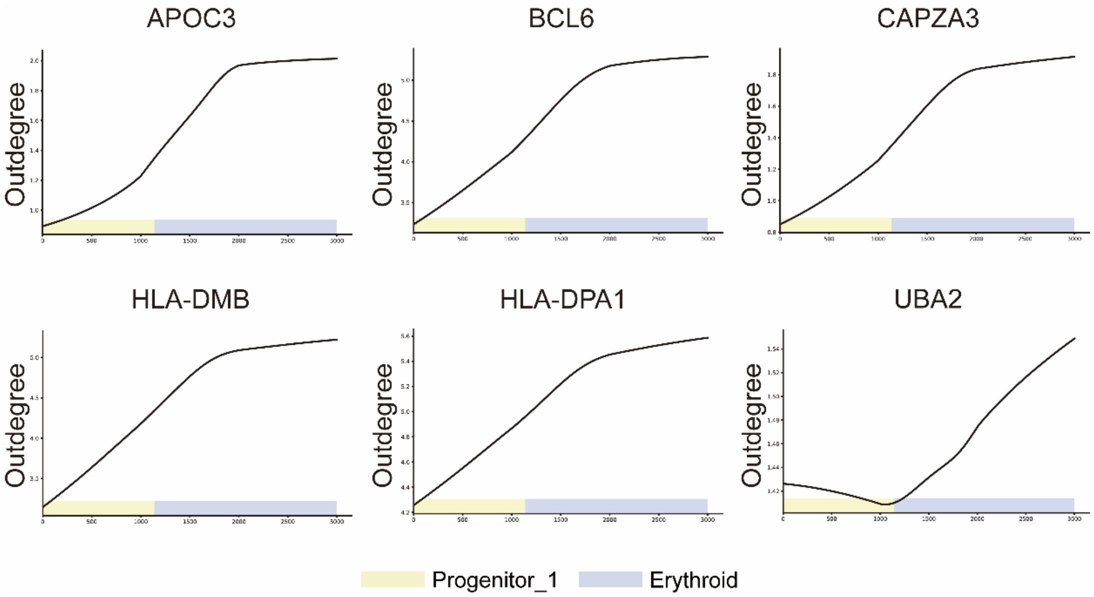

Supplementary Fig. 10. The identified regulators of dynamic changes in Erythroid cell lineage. Examples of regulators with activated patterns of regulatory activity found in erythroid cell lineage. The yellow-violet strip represents the development trajectories of Progenitor-Erythroid lineage.

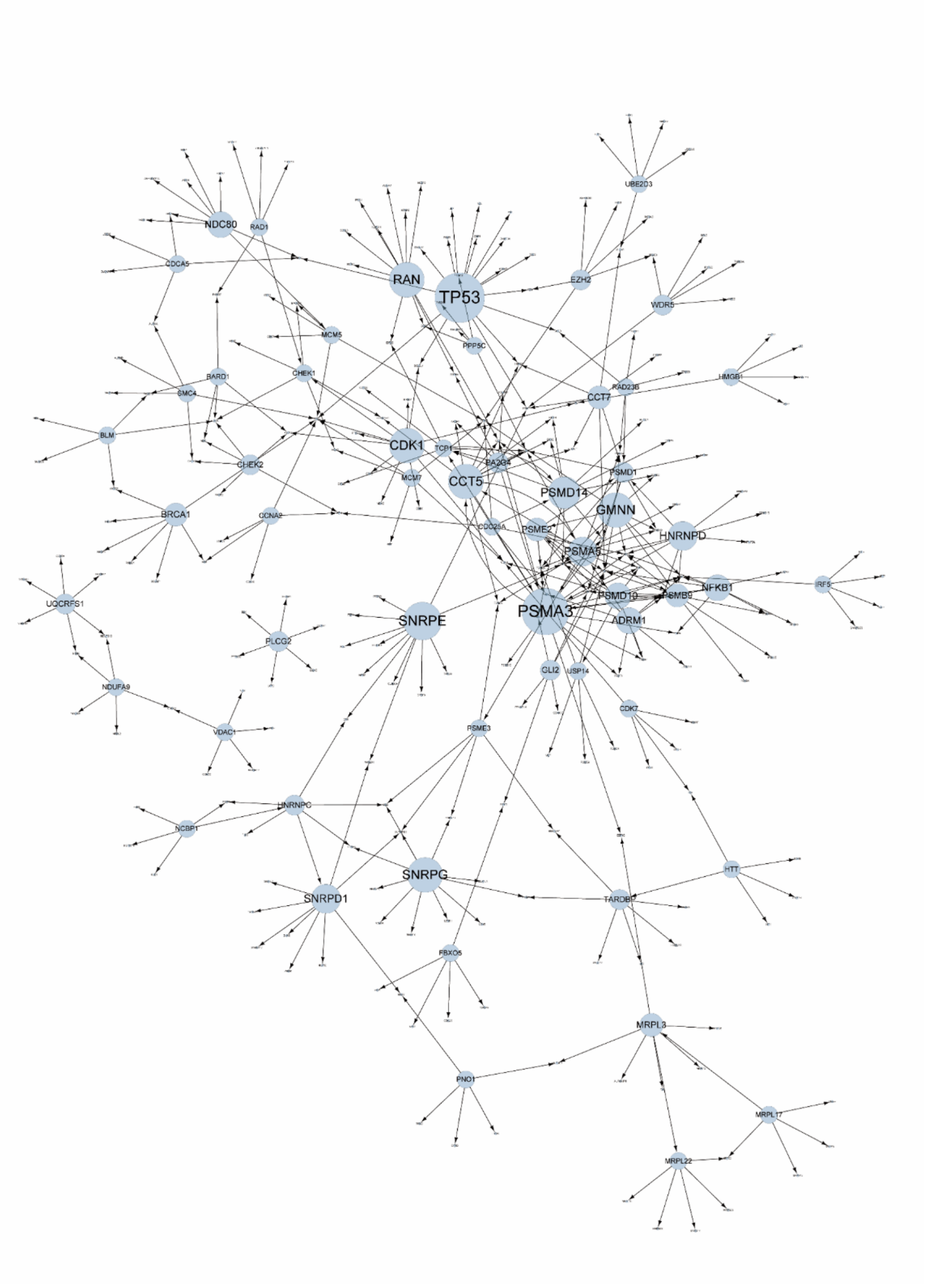

Supplementary Fig. 11. The Progenitor-specific subnetwork in B cell lineage. This subnetwork is composed of key regulators with an outdegree greater than 5, selected from the Progenitor-specific network.

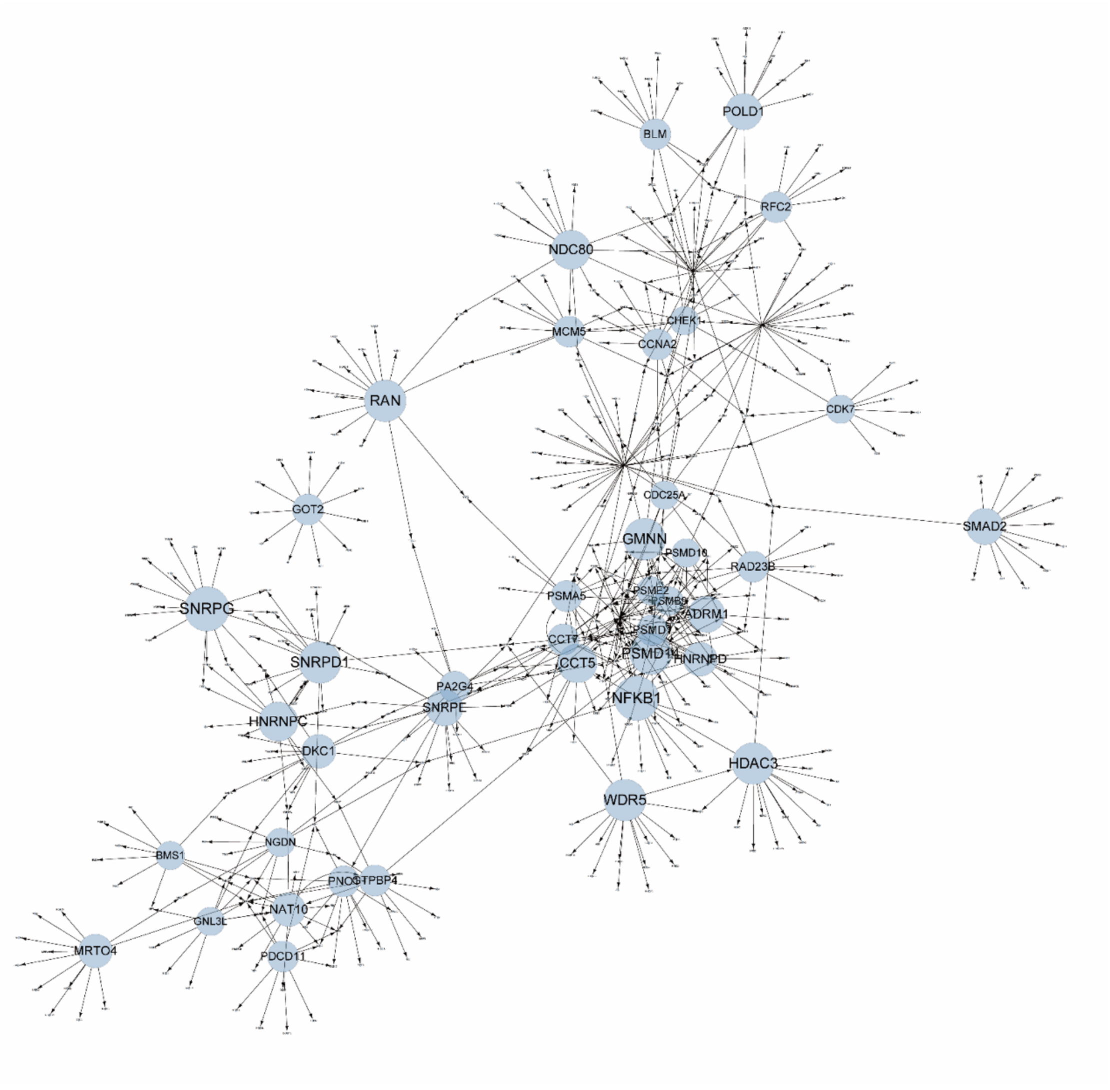

Supplementary Fig. 12. The CLP-specific subnetwork in B cell lineage. This subnetwork is composed of key regulators with an outdegree greater than 10, selected from the CLP-specific network.

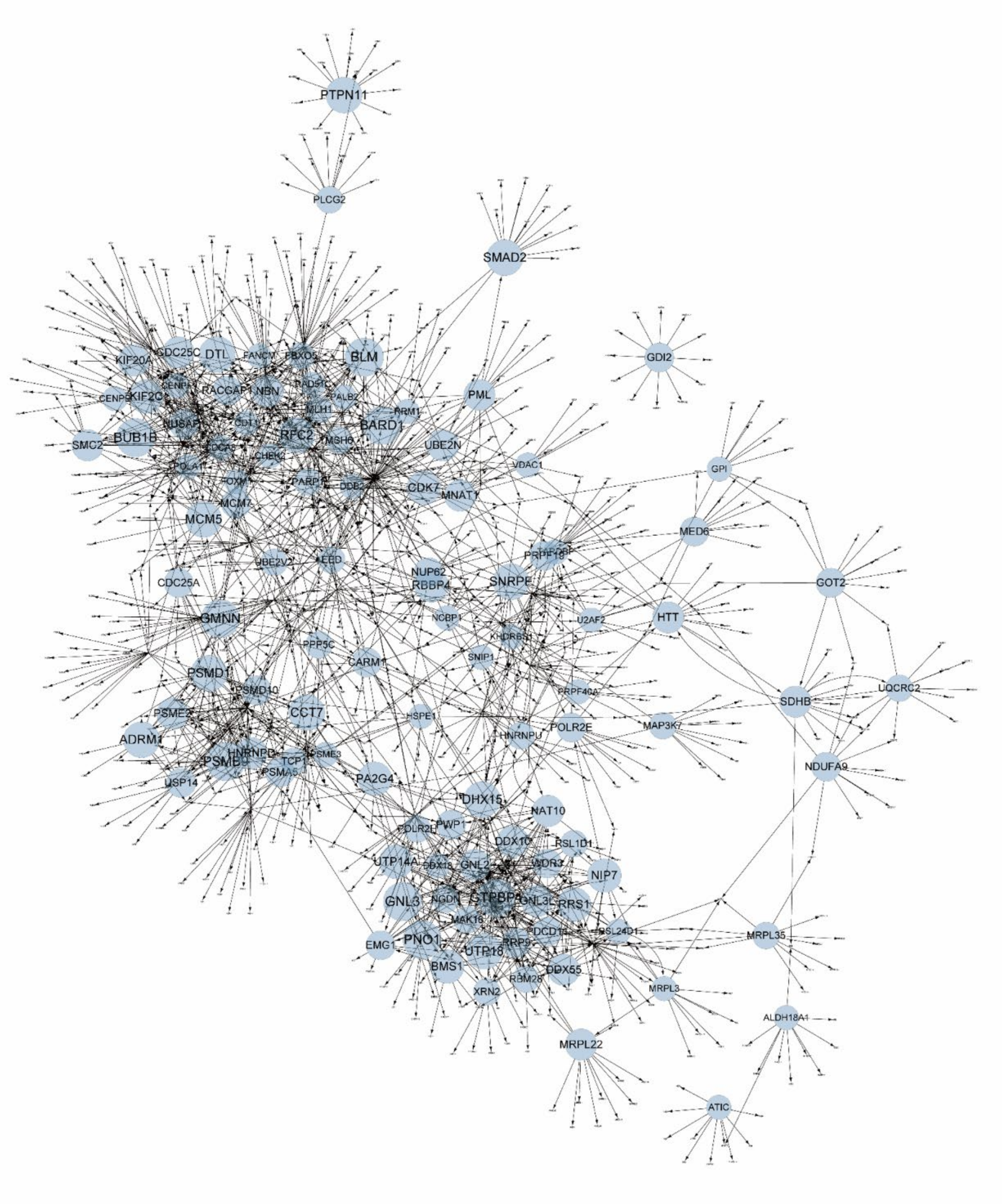

Supplementary Fig. 13. The B cell-specific subnetwork in B cell lineage. This subnetwork is composed of key regulators with an outdegree greater than 10, selected from the B cell-specific network.

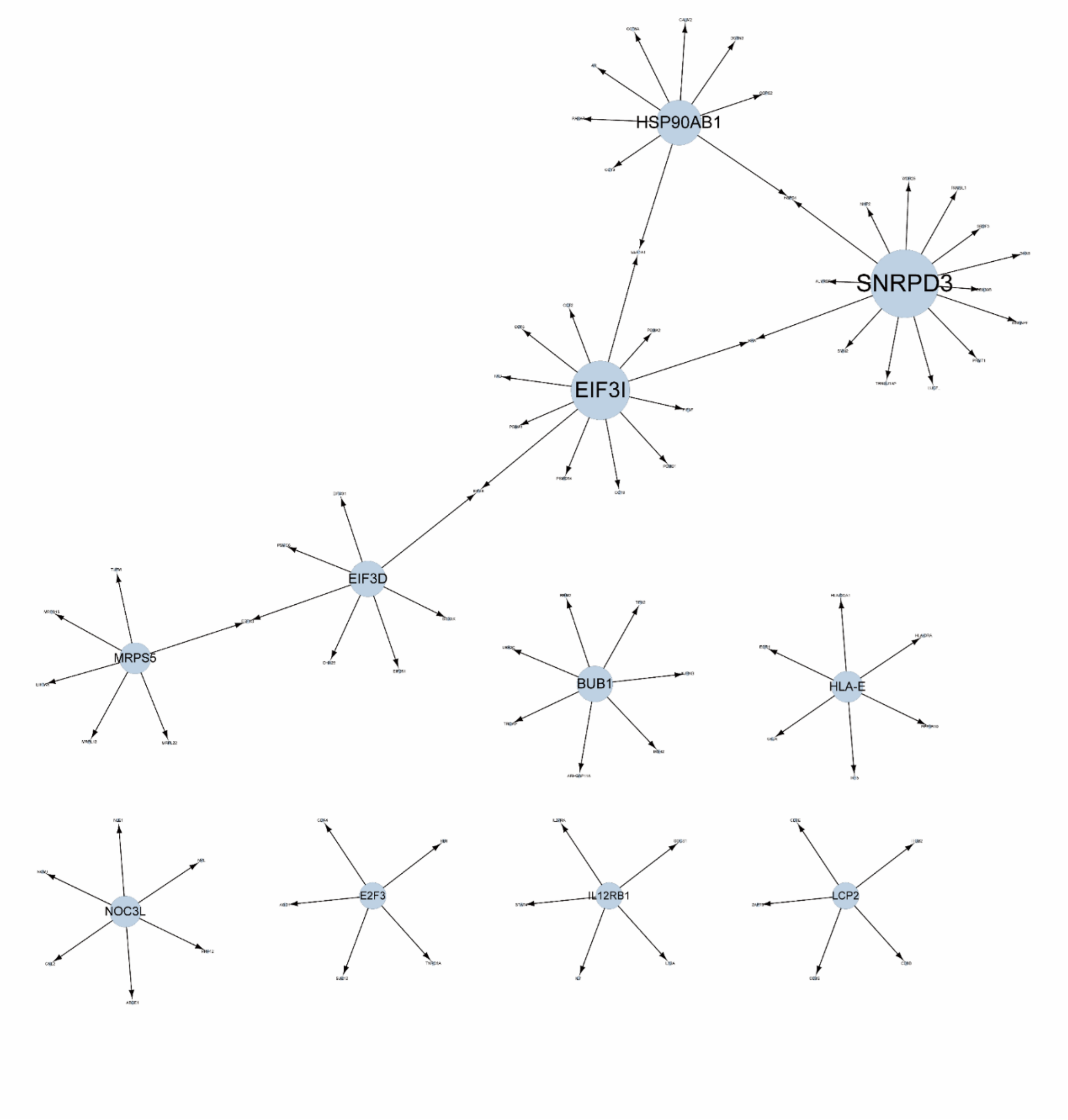

Supplementary Fig. 14. The Progenitor-specific subnetwork in Monocyte cell lineage. This subnetwork is composed of key regulators with an outdegree greater than 5, selected from the progenitor-specific network.

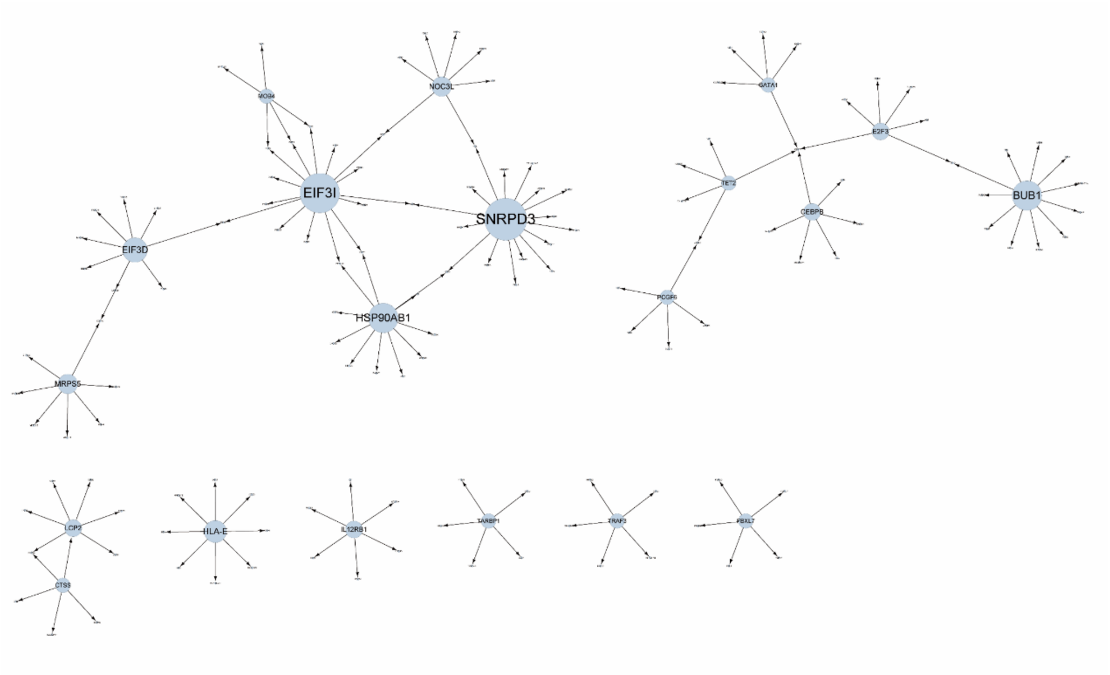

Supplementary Fig. 15. The GMP-specific subnetwork in Monocyte cell lineage. This subnetwork is composed of key regulators with an outdegree greater than 5, selected from the GMP-specific network.

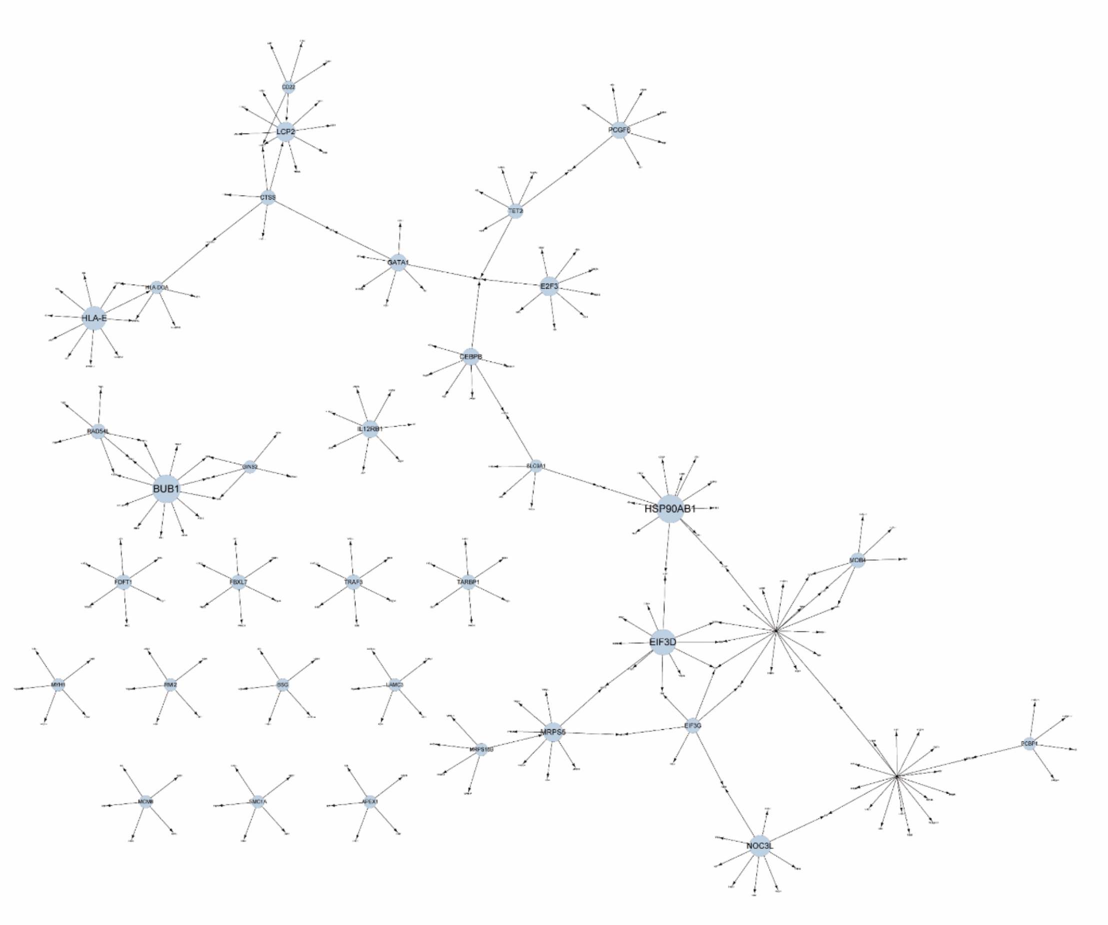

Supplementary Fig. 16. The Monocyte-specific subnetwork in Monocyte cell lineage. This subnetwork is composed of key regulators with an outdegree greater than 5, selected from the Monocyte-specific network.

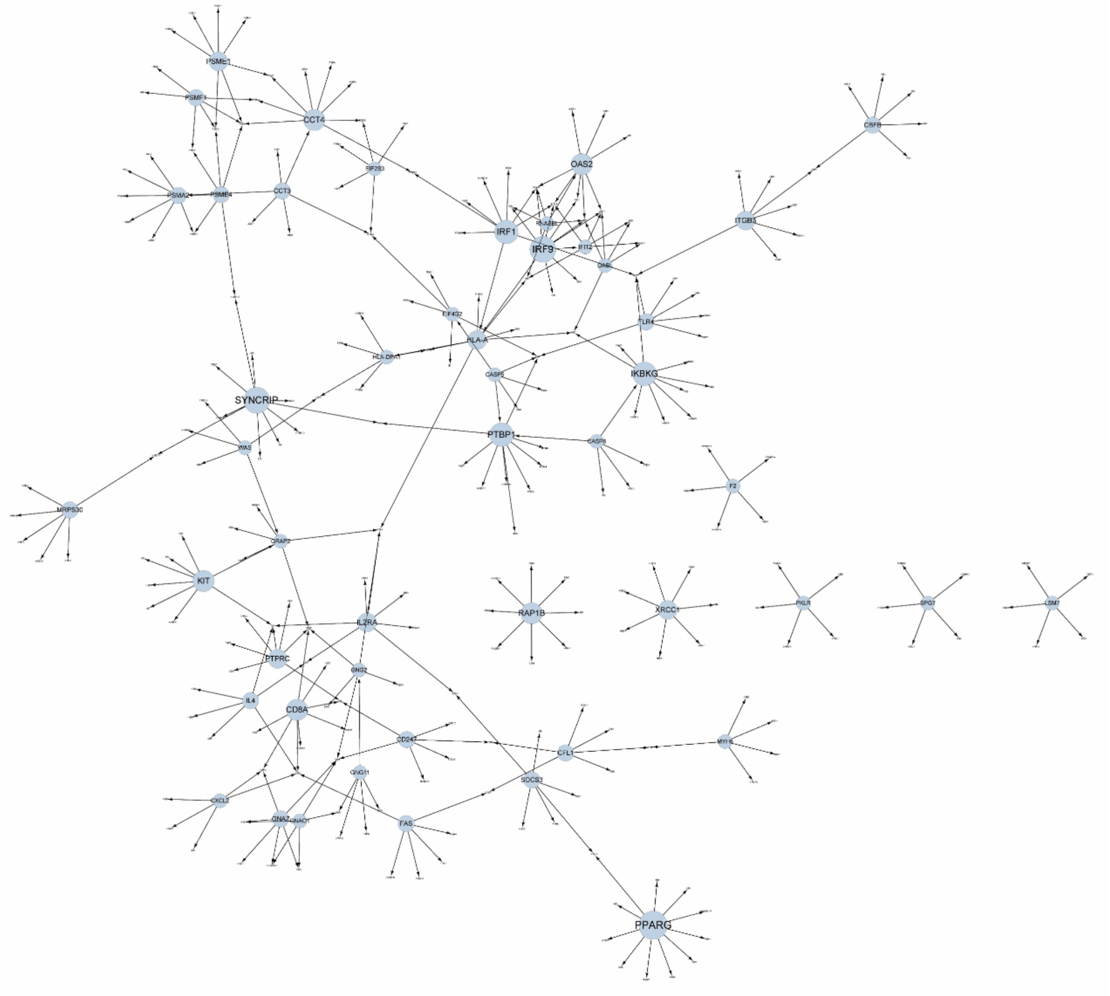

Supplementary Fig. 17. The Progenitor-specific subnetwork in Erythroid cell lineage. This subnetwork is composed of key regulators with an outdegree greater than 5, selected from the Progenitor-specific network.

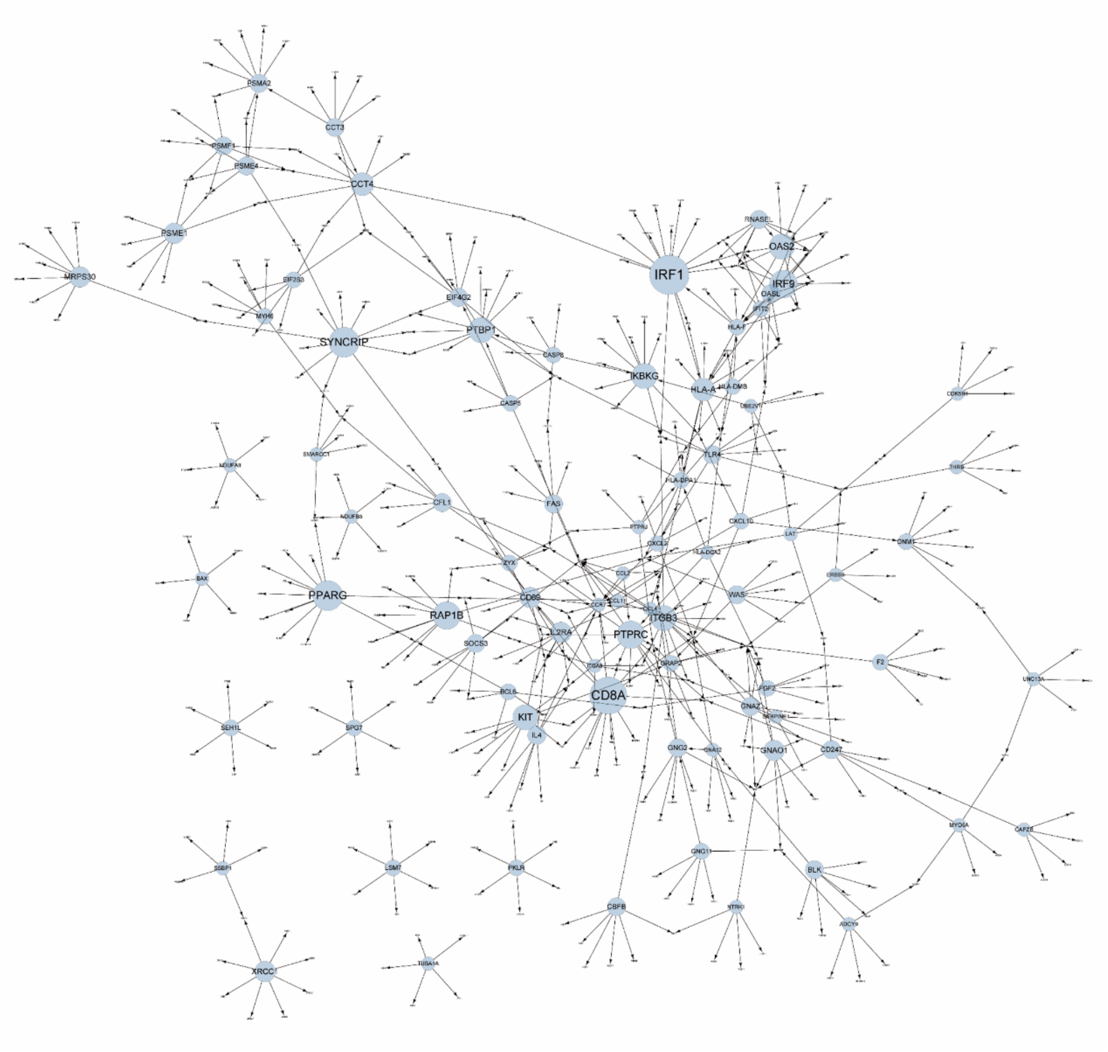

Supplementary Fig. 18. The Erythroid-specific subnetwork in Erythroid cell lineage. This subnetwork is composed of key regulators with an outdegree greater than 5, selected from the Erythroid-specific network.

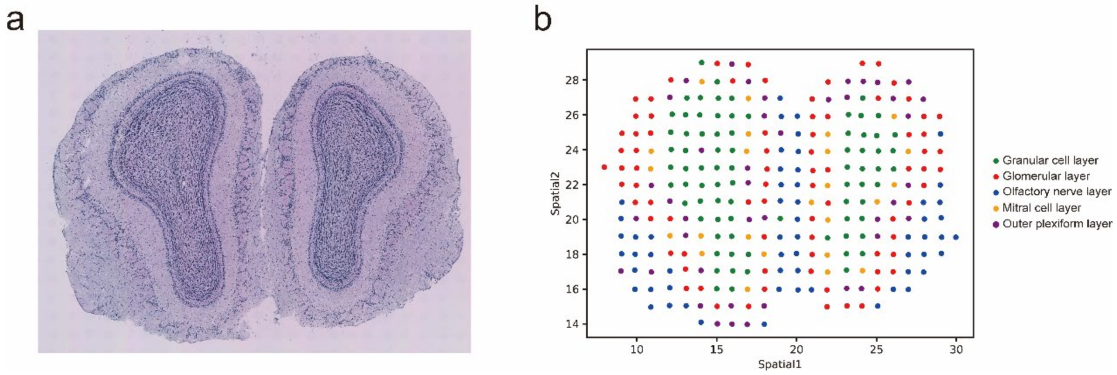

Supplementary Fig. 19. The spatial transcriptomic data of the mouse olfactory bulb. a. The Hematoxylin & eosin stained brightfield image of the mouse olfactory bulb. b. The corresponding tissue domains identified by Leiden.

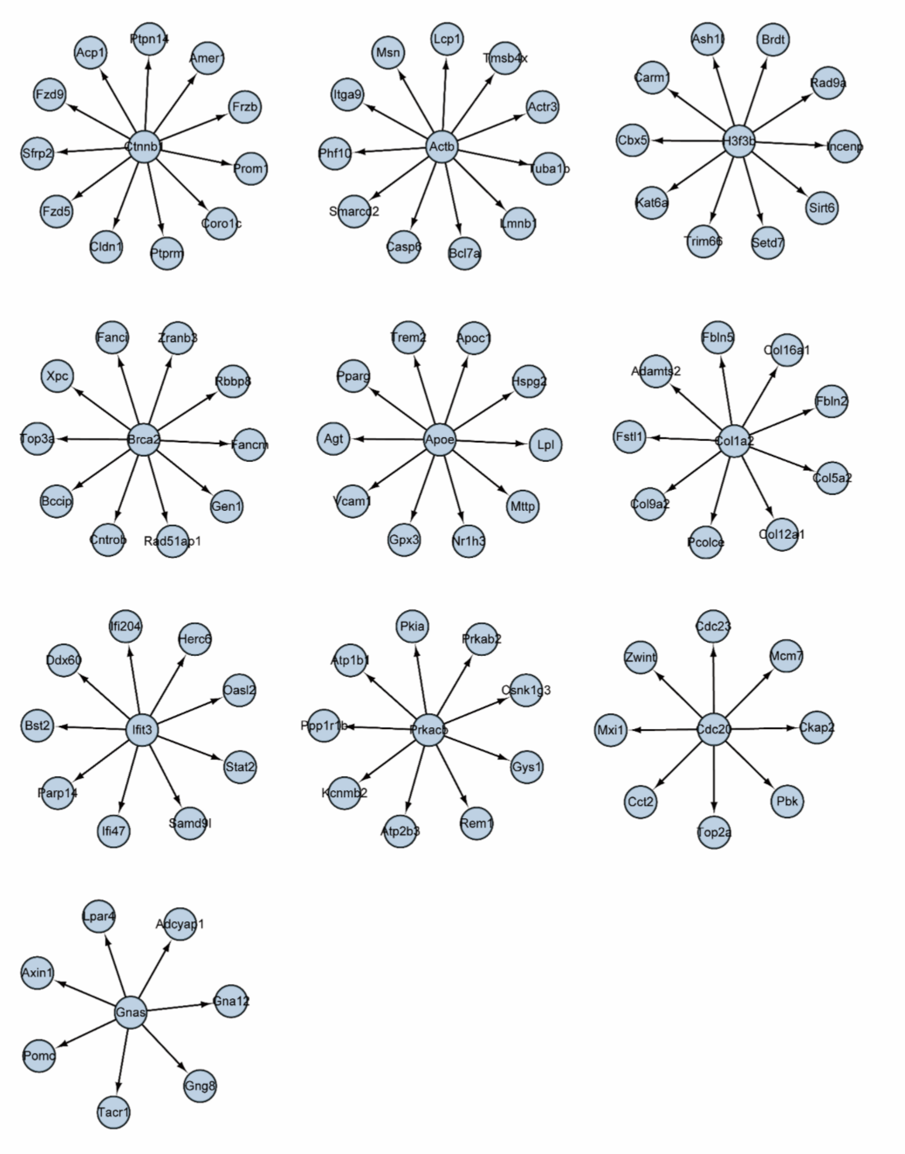

Supplementary Fig. 20. The inferred GL subnetwork in mouse olfactory bulb. This subnetwork is composed of top 10 high regulatory activity regulators, selected from the GL-specific network.

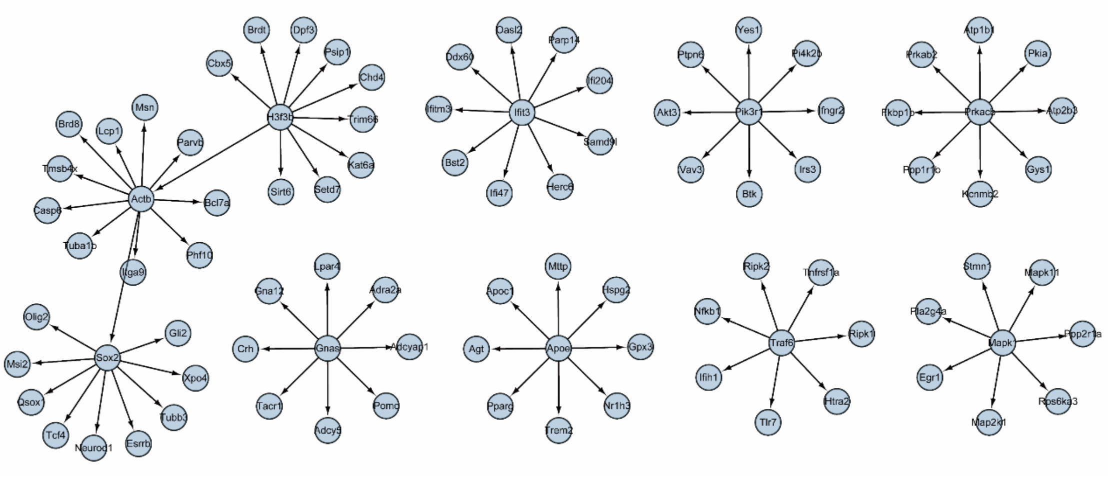

Supplementary Fig. 21. The inferred GCL subnetwork in mouse olfactory bulb. This subnetwork is composed of top 10 high regulatory activity regulators, selected from the GCL-specific network.

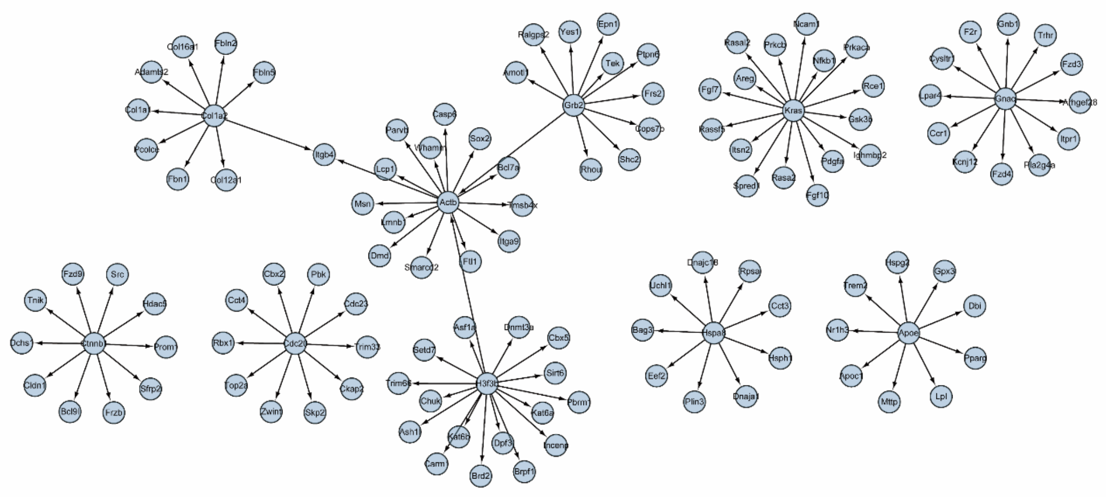

Supplementary Fig. 22. The inferred MCL subnetwork in mouse olfactory bulb. This subnetwork is composed of top 10 high regulatory activity regulators, selected from the MCL-specific network.

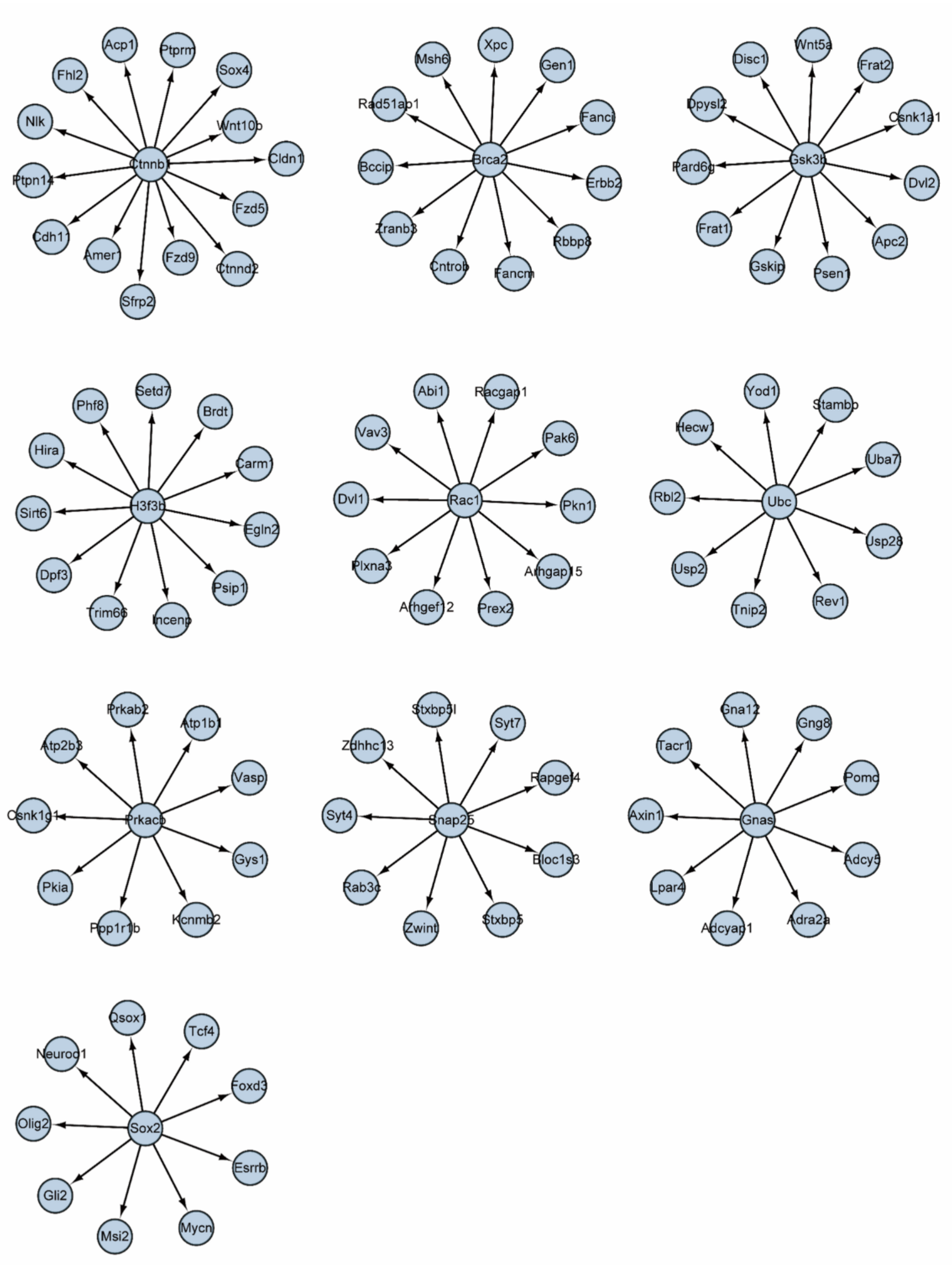

Supplementary Fig. 23. The inferred ONL subnetwork in mouse olfactory bulb. This subnetwork is composed of top 10 high regulatory activity regulators, selected from the ONL-specific network.

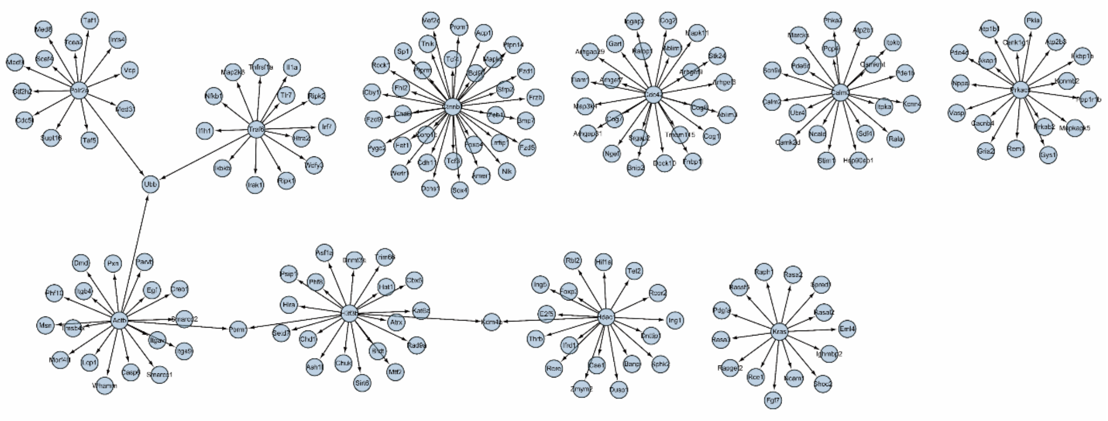

Supplementary Fig. 24. The inferred OPL subnetwork in mouse olfactory bulb. This subnetwork is composed of top 10 high regulatory activity regulators, selected from the OPL-specific network.

Supplementary Tables

Supplementary Tab. 1. The detailed information of five benchmark scRNA-seq datasets.

**Dataset**	**Cells×Genes**	**Cell type**	**Normalization**	**Source**
**Biase**	**49×11470**	**3**	**FPKM**	**GSE57249**
**Enge**	**2282×12118**	**6**	**CPM**	**GSE81547**
**Darmanis**	**331×11480**	**7**	**CPM**	**GSE67835**
**Fink**	**3045×13090**	**7**	**CPM**	**GSE184111**
**Goolam**	**124×11178**	**5**	**CPM**	**E-MTAB-3321**

Supplementary Tab. 2. The results of 26 identified key regulators with FDR-adjusted *p* values from ODM.

Key regulator	Normal vs Border	Normal vs Core	Border vs Core
ABL1	5.36×10^{-108}	2.20×10^{-219}	4.51×10^{-45}
PPARA	4.31×10^{-35}	4.25×10^{-116}	3.36×10^{-42}
PPARG	2.32×10^{-240}	$<1.00\times10^{-314}$	3.15×10^{-53}
NCOA1	1.67×10^{-34}	6.07×10^{-157}	2.49×10^{-100}
ATF3	4.25×10^{-73}	4.89×10^{-278}	1.16×10^{-149}
LMX1B	9.64×10^{-233}	$<1.00\times10^{-314}$	2.08×10^{-106}
PPP2R3B	6.34×10^{-185}	$<1.00\times10^{-314}$	1.77×10^{-278}
IVD	5.01×10^{-146}	1.39×10^{-314}	2.64×10^{-135}
NR3C2	1.60×10^{-294}	$<1.00\times10^{-314}$	9.53×10^{-224}
TCF7	1.15×10^{-101}	$<1.00\times10^{-314}$	2.52×10^{-242}
NR3C1	5.86×10^{-248}	$<1.00\times10^{-314}$	2.22×10^{-232}
ECSIT	$<1.00\times10^{-314}$	$<1.00\times10^{-314}$	5.14×10^{-116}
POU5F1	$<1.00\times10^{-314}$	$<1.00\times10^{-314}$	$<1.00\times10^{-314}$
JUN	$<1.00\times10^{-314}$	$<1.00\times10^{-314}$	$<1.00\times10^{-314}$
KLF4	$<1.00\times10^{-314}$	$<1.00\times10^{-314}$	2.49×10^{-83}
EGR1	$<1.00\times10^{-314}$	$<1.00\times10^{-314}$	$<1.00\times10^{-314}$
FOSB	$<1.00\times10^{-314}$	$<1.00\times10^{-314}$	$<1.00\times10^{-314}$
SUCLG1	$<1.00\times10^{-314}$	$<1.00\times10^{-314}$	8.63×10^{-189}
GOT1	$<1.00\times10^{-314}$	$<1.00\times10^{-314}$	8.42×10^{-215}
PCK2	$<1.00\times10^{-314}$	$<1.00\times10^{-314}$	$<1.00\times10^{-314}$
XBP1	$<1.00\times10^{-314}$	$<1.00\times10^{-314}$	$<1.00\times10^{-314}$
NCOR1	$<1.00\times10^{-314}$	$<1.00\times10^{-314}$	$<1.00\times10^{-314}$
EGR2	$<1.00\times10^{-314}$	$<1.00\times10^{-314}$	$<1.00\times10^{-314}$
MLXIP	$<1.00\times10^{-314}$	$<1.00\times10^{-314}$	$<1.00\times10^{-314}$
ACAA1	$<1.00\times10^{-314}$	$<1.00\times10^{-314}$	$<1.00\times10^{-314}$
HNF4A	$<1.00\times10^{-314}$	$<1.00\times10^{-314}$	$<1.00\times10^{-314}$

The FDR-adjusted *p* values of 26 identified key regulators calculated by ODM are presented. ‘Normal vs Border’ represents the comparison between ODM data from the healthy adjacent region (Normal) and the border region (Border). ‘Normal vs Core’ represents the comparison between ODM data from the healthy adjacent region (Normal) and the core region (Core). ‘Border vs Core’ represents the comparison between ODM data from the border region (Border) and the core region (Core).

Supplementary Tab. 3. The results of 26 identified key regulators with FDR-adjusted *p* values from GEM.

Key regulator	Normal vs Border	Normal vs Core	Border vs Core
ABL1	8.04×10^{-3}	1.85×10^{-5}	1.47×10^{-2}
PPARA	**0.295**	2.62×10^{-2}	1.80×10^{-5}
PPARG	2.69×10^{-11}	8.44×10^{-11}	**0.474**
NCOA1	**0.828**	**0.507**	**0.569**
ATF3	1.98×10^{-6}	3.14×10^{-23}	2.62×10^{-11}
LMX1B	2.44×10^{-2}	2.59×10^{-2}	**0.987**
PPP2R3B	1.45×10^{-2}	**0.639**	1.69×10^{-2}
IVD	8.76×10^{-9}	6.24×10^{-9}	**0.616**
NR3C2	5.23×10^{-35}	1.85×10^{-28}	**0.942**
TCF7	**0.383**	8.18×10^{-3}	9.42×10^{-6}
NR3C1	7.14×10^{-11}	3.77×10^{-8}	**0.597**
ECSIT	7.14×10^{-11}	2.50×10^{-10}	**0.573**
POU5F1	**0.942**	**0.522**	8.92×10^{-3}
JUN	1.75×10^{-78}	8.84×10^{-101}	6.95×10^{-7}
KLF4	8.52×10^{-157}	1.09×10^{-183}	6.98×10^{-9}
EGR1	1.32×10^{-47}	1.06×10^{-90}	8.83×10^{-15}
FOSB	2.25×10^{-31}	8.85×10^{-67}	1.56×10^{-13}
SUCLG1	1.06×10^{-91}	1.79×10^{-97}	9.16×10^{-4}
GOT1	1.35×10^{-12}	9.94×10^{-14}	**0.156**
PCK2	1.81×10^{-22}	3.27×10^{-30}	9.16×10^{-4}
XBP1	6.22×10^{-14}	1.68×10^{-27}	1.59×10^{-7}
NCOR1	3.30×10^{-7}	3.87×10^{-21}	9.37×10^{-10}
EGR2	3.29×10^{-3}	**0.0876**	**0.216**
MLXIP	1.24×10^{-28}	2.22×10^{-37}	8.04×10^{-4}
ACAA1	2.25×10^{-23}	2.41×10^{-29}	5.47×10^{-3}
HNF4A	5.79×10^{-3}	9.50×10^{-10}	3.80×10^{-6}

The FDR-adjusted *p* values of 26 identified key regulators calculated by GEM are presented, where those marked in bold indicate non-significant *p* values, specifically those greater than 0.05. ‘Normal vs Border’ represents the comparison between GEM data from the healthy adjacent region (Normal) and the border region (Border). ‘Normal vs Core’ represents the comparison between GEM data from the healthy adjacent region (Normal) and the core region (Core). ‘Border vs Core’ represents the comparison between GEM data from the border region (Border) and the core region (Core).

Supplementary Tab. 4. The chi-square test and FDR-adjusted *p* values for identified significant upregulation links.

Upregulation links	chi2_pvalue	FDR_corrected_pvalue
KLF4_NR5A2	2.89×10^{-145}	3.84×10^{-143}
POU5F1_CDX2	6.44×10^{-143}	4.28×10^{-141}
KLF4_MYCL	1.54×10^{-120}	6.82×10^{-119}
KLF4_TBX3	3.39×10^{-103}	1.13×10^{-101}
KLF4_TCF4	1.96×10^{-95}	5.23×10^{-94}
JUN_CDK5R1	8.25×10^{-87}	1.83×10^{-85}
JUN_STAT5A	3.49×10^{-77}	6.63×10^{-76}
NR3C1_HSD11B2	2.98×10^{-67}	4.95×10^{-66}
JUN_NKRF	4.64×10^{-60}	6.85×10^{-59}
PPARG_PCK1	3.85×10^{-58}	5.13×10^{-57}
LMX1B_MEIS1	3.97×10^{-57}	4.80×10^{-56}
JUN_DET1	9.59×10^{-56}	1.06×10^{-54}
PCK2_PCK1	2.82×10^{-54}	2.88×10^{-53}
JUN_BATF2	8.68×10^{-54}	8.24×10^{-53}
SUCLG1_TXNRD2	3.25×10^{-53}	2.88×10^{-52}
SUCLG1_RNF138	3.35×10^{-52}	2.79×10^{-51}
JUN_CXCL2	8.83×10^{-52}	6.91×10^{-51}
KLF4_PBX1	6.44×10^{-50}	4.76×10^{-49}
NR3C2_HSD11B2	1.48×10^{-48}	1.04×10^{-47}
SUCLG1_ACACB	4.51×10^{-48}	3.00×10^{-47}
NR3C2_SCNN1B	7.83×10^{-48}	4.96×10^{-47}
EGR2_ID2	1.66×10^{-45}	1.00×10^{-44}
JUN_FBXW7	1.31×10^{-44}	7.59×10^{-44}
JUN_NCOA6	3.28×10^{-43}	1.82×10^{-42}
KLF4_HDAC3	2.04×10^{-42}	1.09×10^{-41}
JUN_MAP3K5	1.26×10^{-41}	6.47×10^{-41}
EGR1_SRF	2.71×10^{-39}	1.33×10^{-38}
JUN_RPS6KB1	4.50×10^{-39}	2.14×10^{-38}
KLF4_CREBBP	5.11×10^{-38}	2.34×10^{-37}
JUN_MECOM	1.18×10^{-32}	5.21×10^{-32}
EGR1_NR3C1	3.50×10^{-32}	1.50×10^{-31}
JUN_CTNNB1	6.77×10^{-32}	2.81×10^{-31}
JUN_CRBN	1.35×10^{-31}	5.45×10^{-31}
JUN_FOXA1	6.03×10^{-31}	2.36×10^{-30}
JUN_NRIP1	7.75×10^{-31}	2.94×10^{-30}
JUN_CSK	1.01×10^{-28}	3.72×10^{-28}
PPARA_HMGCS2	2.66×10^{-27}	9.54×10^{-27}
JUN_MAP2K7	2.14×10^{-26}	7.51×10^{-26}
JUN_HSPA4	4.11×10^{-26}	1.40×10^{-25}

ATF3_CHAC1	1.65×10^{-25}	5.49×10^{-25}
ACAA1_HMGCS2	2.86×10^{-25}	9.28×10^{-25}
TCF7_CTNNB1	4.63×10^{-25}	1.47×10^{-24}
HNF4A_CTNNB1	2.11×10^{-22}	6.51×10^{-22}
JUN_CDKN1A	1.63×10^{-21}	4.94×10^{-21}
IVD_ECI2	5.16×10^{-21}	1.52×10^{-20}
FOSB_MAF	3.71×10^{-20}	1.07×10^{-19}
KLF4_ESRRA	5.69×10^{-20}	1.61×10^{-19}
NCOR1_SMAD4	6.33×10^{-19}	1.75×10^{-18}
NCOR1_SKI	3.36×10^{-18}	9.11×10^{-18}
EGR1_ELK4	2.92×10^{-17}	7.77×10^{-17}
XBP1_TSPYL2	3.24×10^{-17}	8.44×10^{-17}
EGR2_NAB1	7.15×10^{-17}	1.83×10^{-16}
LMX1B_NR4A2	7.68×10^{-17}	1.93×10^{-16}
EGR1_DUSP1	1.81×10^{-16}	4.46×10^{-16}
NCOR1_PATZ1	2.84×10^{-16}	6.87×10^{-16}
NCOR1_NR2C2	2.07×10^{-15}	4.92×10^{-15}
ACAA1_HADH	3.34×10^{-15}	7.80×10^{-15}
LMX1B_PBX2	9.96×10^{-15}	2.28×10^{-14}
EGR1_NAB1	1.85×10^{-14}	4.17×10^{-14}
NCOR1_NR6A1	2.87×10^{-13}	6.37×10^{-13}
ACAA1_ACADS	3.51×10^{-13}	7.66×10^{-13}
MLXIP_MLXIPL	4.36×10^{-13}	9.35×10^{-13}
XBP1_WIPI1	2.37×10^{-12}	5.01×10^{-12}
EGR1_TCF12	5.37×10^{-12}	1.12×10^{-11}
POU5F1_GFER	6.11×10^{-12}	1.25×10^{-11}
NCOR1_ETV6	6.41×10^{-12}	1.29×10^{-11}
PPP2R3B_GTPBP6	1.40×10^{-11}	2.79×10^{-11}
HNF4A_SMAD7	2.27×10^{-11}	4.44×10^{-11}
ABL1_CCND2	2.59×10^{-11}	5.00×10^{-11}
FOSB_MAFG	3.04×10^{-11}	5.77×10^{-11}
NCOR1_NR2C1	3.53×10^{-11}	6.61×10^{-11}
ATF3_MAFF	5.52×10^{-11}	1.02×10^{-10}
NCOR1_NRIP1	6.60×10^{-11}	1.20×10^{-10}
NCOR1_NR2F2	8.30×10^{-11}	1.49×10^{-10}
MLXIP_PPARGC1B	9.75×10^{-11}	1.73×10^{-10}
ACAA1_PECR	1.17×10^{-10}	2.04×10^{-10}
NCOR1_TAB2	1.45×10^{-10}	2.50×10^{-10}
NCOR1_BDP1	1.98×10^{-10}	3.38×10^{-10}
NCOR1_HDAC11	2.27×10^{-10}	3.82×10^{-10}
MLXIP_MXD4	4.31×10^{-10}	7.16×10^{-10}
HNF4A_FOXA2	5.57×10^{-10}	9.04×10^{-10}
ATF3_ATF2	5.57×10^{-10}	9.04×10^{-10}

ABL1_SORBS2	1.22×10^{-9}	1.95×10^{-9}
ABL1_NTRK2	1.26×10^{-9}	2.00×10^{-9}
GOT1_GPT2	1.92×10^{-9}	3.00×10^{-9}
PPP2R3B_PPP4R2	2.16×10^{-9}	3.34×10^{-9}
XBP1_HERPUD1	3.40×10^{-9}	5.20×10^{-9}
NCOA1_NR4A1	3.55×10^{-9}	5.36×10^{-9}
ABL1_CDKN1B	3.77×10^{-9}	5.64×10^{-9}
NR3C2_APEH	6.86×10^{-9}	1.01×10^{-8}
NCOR1_SMARCA2	7.61×10^{-9}	1.11×10^{-8}
PPARG_RXRB	1.38×10^{-8}	2.00×10^{-8}
ABL1_CRK	1.74×10^{-8}	2.49×10^{-8}
PPARA_GPT	1.82×10^{-8}	2.57×10^{-8}
FOSB_EGR1	2.12×10^{-8}	2.96×10^{-8}
IVD_ACAA1	2.67×10^{-8}	3.70×10^{-8}
PCK2_GOT1	4.76×10^{-8}	6.53×10^{-8}
PPARG_PLIN2	1.53×10^{-7}	2.07×10^{-7}
PPARG_ESRRA	2.66×10^{-7}	3.57×10^{-7}
PPARA_PDK4	3.62×10^{-7}	4.82×10^{-7}
NCOA1_NR3C2	4.39×10^{-7}	5.78×10^{-7}
TCF7_SOX4	6.14×10^{-7}	8.00×10^{-7}
ACAA1_PEX13	6.68×10^{-7}	8.63×10^{-7}
PPP2R3B_PPP2R3A	1.26×10^{-6}	1.62×10^{-6}
PPARA_PCK2	1.40×10^{-6}	1.77×10^{-6}
XBP1_ERN2	1.78×10^{-6}	2.23×10^{-6}
ABL1_RAC3	5.57×10^{-6}	6.92×10^{-6}
HNF4A_GATA6	5.93×10^{-6}	7.30×10^{-6}
ECSIT_TMEM186	8.65×10^{-6}	1.06×10^{-5}
HNF4A_TCF7	9.57×10^{-6}	1.16×10^{-5}
NCOR1_PPARA	9.65×10^{-6}	1.16×10^{-5}
GOT1_PCK2	1.04×10^{-5}	1.24×10^{-5}
IVD_EHHADH	2.43×10^{-5}	2.86×10^{-5}
NCOR1_SPEN	2.48×10^{-5}	2.90×10^{-5}
ATF3_FOSB	5.15×10^{-5}	5.96×10^{-5}
FOSB_TRIB1	9.35×10^{-5}	1.07×10^{-4}
NR3C1_DAXX	1.31×10^{-4}	1.49×10^{-4}
ECSIT_GCAT	2.28×10^{-4}	2.57×10^{-4}
PPARG_PAX8	3.06×10^{-4}	3.42×10^{-4}
PPARA_PPIF	3.10×10^{-4}	3.44×10^{-4}
HNF4A_PER2	3.17×10^{-4}	3.48×10^{-4}
EGR1_DUSP6	3.80×10^{-4}	4.14×10^{-4}
GOT1_SULT1A3	3.83×10^{-4}	4.14×10^{-4}
ACAA1_PHYH	6.05×10^{-4}	6.49×10^{-4}
PPARG_SREBF2	8.94×10^{-4}	9.52×10^{-4}

ATF3_GADD45B	1.11×10^{-3}	1.17×10^{-3}
FOSB_ATF3	1.22×10^{-3}	1.28×10^{-3}
POU5F1_FOXA1	1.75×10^{-3}	1.82×10^{-3}
IVD_PCCA	2.06×10^{-3}	2.12×10^{-3}
PPARG_KDM3A	3.94×10^{-3}	4.03×10^{-3}
ECSIT_MGME1	4.77×10^{-3}	4.85×10^{-3}
PPARG_MAP2K1	1.51×10^{-2}	1.53×10^{-2}
NCOA1_NCOA6	2.15×10^{-2}	2.15×10^{-2}

Supplementary Tab. 5. The detailed ODM for each cell during B cell lineage.

The table is an Excel file, please access it on below URL:
https://github.com/Huang-XZ-Sandy/SiCNet/tree/main/Supplementary%20Table

Supplementary Tab. 6. The detailed ODM for each cell during Monocyte cell lineage.

The table is an Excel file, please access it on below URL:
https://github.com/Huang-XZ-Sandy/SiCNet/tree/main/Supplementary%20Table

Supplementary Tab. 7. The detailed ODM for each cell during Erythroid cell lineage.

The table is an Excel file, please access it on below URL:
https://github.com/Huang-XZ-Sandy/SiCNet/tree/main/Supplementary%20Table

Supplementary Tab. 8. The running time of SiCNet using four datasets containing reference data of different sizes

Dataset	Reference data size (Cells×Genes)	Reference network running time (Hours)	One cell network running time (Minutes)
Goolam	7×17195	0.276	3.061
Enge	262×17168	5.058	9.709
CRC	1144×17056	14.257	10.640
Fink	2000×19231	17.919	16.726

Reference paper

[1] X. Liu, X. Chang, S. Leng, H. Tang, K. Aihara, L. Chen, Detection for disease tipping points by landscape dynamic network biomarkers, National science review 6(4) (2019) 775-785.
[2] F.A. Wolf, P. Angerer, F.J. Theis, SCANPY: large-scale single-cell gene expression data analysis, Genome biology 19 (2018) 1-5.
[3] K. Street, D. Risso, R.B. Fletcher, D. Das, J. Ngai, N. Yosef, E. Purdom, S. Dudoit, Slingshot: cell lineage and pseudotime inference for single-cell transcriptomics, BMC genomics 19 (2018) 1-16.
[4] P. Shannon, A. Markiel, O. Ozier, N.S. Baliga, J.T. Wang, D. Ramage, N. Amin, B. Schwikowski, T. Ideker, Cytoscape: a software environment for integrated models of biomolecular interaction networks, Genome research 13(11) (2003) 2498-2504.
[5] J.M. Granja, S. Klemm, L.M. McGinnis, A.S. Kathiria, A. Mezger, M.R. Corces, B. Parks, E. Gars, M. Liedtke, G.X. Zheng, Single-cell multiomic analysis identifies regulatory programs in mixed-phenotype acute leukemia, Nature biotechnology 37(12) (2019) 1458-1465.
[6] L. Wang, N. Trasanidis, T. Wu, G. Dong, M. Hu, D.E. Bauer, L. Pinello, Dictys: dynamic gene regulatory network dissects developmental continuum with single-cell multiomics, Nature Methods 20(9) (2023) 1368-1378.
[7] J.-H. Lee, ATM in immunobiology: From lymphocyte development to cancer immunotherapy, Translational Oncology 52 (2025) 102268.
[8] P. Muralidhara, A. Kumar, M.K. Chaurasia, K. Bansal, Topoisomerases in immune cell development and function, The Journal of Immunology 210(2) (2023) 126-133.
[9] J. Korzhenevich, I. Janowska, M. van der Burg, M. Rizzi, Human and mouse early B cell development: So similar but so different, Immunology Letters 261 (2023) 1-12.
[10] S. Sergejeva, A. Linden, Impact of IL-17 on cells of the monocyte lineage in health and disease, Endocrine, Metabolic & Immune Disorders-Drug Targets (Formerly Current Drug Targets-Immune, Endocrine & Metabolic Disorders) 9(2) (2009) 178-186.
[11] F. Al-Rashed, Z. Ahmad, A.J. Snider, R. Thomas, S. Kochumon, M. Melhem, S. Sindhu, L.M. Obeid, F. Al-Mulla, Y.A. Hannun, Ceramide kinase regulates TNF-α-induced immune responses in human monocytic cells, Scientific Reports 11(1) (2021) 8259.
[12] E. Dzierzak, S. Philipsen, Erythropoiesis: development and differentiation, Cold Spring Harbor perspectives in medicine 3(4) (2013) a011601.
[13] P.L. Ståhl, F. Salmén, S. Vickovic, A. Lundmark, J.F. Navarro, J. Magnusson, S. Giacomello, M. Asp, J.O. Westholm, M. Huss, Visualization and analysis of gene expression in tissue sections by spatial transcriptomics, Science 353(6294) (2016) 78-82.
[14] I. Nwosu, S. Gairhe, R.G. Struble, B.P. Nathan, Impact of apoE deficiency during synaptic remodeling in the mouse olfactory bulb, Neuroscience letters 441(3) (2008) 282-285.
[15] B.S. East, G. Fleming, K. Peng, J.K. Olofsson, E. Levy, P.M. Mathews, D.A. Wilson, Human apolipoprotein E genotype differentially affects olfactory behavior and sensory physiology in mice, Neuroscience 380 (2018) 103-110.
[16] J. Zhang, C. Hao, J. Jiang, Y. Feng, X. Chen, Y. Zheng, J. Liu, Z. Zhang, C. Long, L. Yang, The mechanisms underlying olfactory deficits in apolipoprotein E–deficient mice: focus on olfactory epithelium and olfactory bulb, Neurobiology of Aging 62 (2018) 20-33.
[17] Z. Xu, L. Wang, G. Chen, X. Rao, F. Xu, Roles of GSK3β in odor habituation and spontaneous

neural activity of the mouse olfactory bulb, PLoS One 8(5) (2013) e63598.
[18] Q. Zhang, W. Yan, Y. Bai, Y. Zhu, J. Ma, Repeated formaldehyde inhalation impaired olfactory function and changed SNAP25 proteins in olfactory bulb, International Journal of Occupational and Environmental Health 20(4) (2014) 308-312.
[19] V.Y. Kiselev, K. Kirschner, M.T. Schaub, T. Andrews, A. Yiu, T. Chandra, K.N. Natarajan, W. Reik, M. Barahona, A.R. Green, SC3: consensus clustering of single-cell RNA-seq data, Nature methods 14(5) (2017) 483-486.
[20] P. Lin, M. Troup, J.W. Ho, CIDR: Ultrafast and accurate clustering through imputation for single-cell RNA-seq data, Genome biology 18 (2017) 1-11.
[21] B. Wang, J. Zhu, E. Pierson, D. Ramazzotti, S. Batzoglou, Visualization and analysis of single-cell RNA-seq data by kernel-based similarity learning, Nature methods 14(4) (2017) 414-416.
[22] M. Bilous, L. Tran, C. Cianciaruso, A. Gabriel, H. Michel, S.J. Carmona, M.J. Pittet, D. Gfeller, Metacells untangle large and complex single-cell transcriptome networks, BMC bioinformatics 23(1) (2022) 336.
